# A global analysis of adaptation to societal aging across low-, middle- and high-income countries using the Global Aging Society Index

**DOI:** 10.1038/s43587-024-00772-3

**Published:** 2024-12-27

**Authors:** Cynthia Chen, Julian Lim, Jemima Koh, John Beard, John W. Rowe, for the Research Network on an Aging Society, Cynthia Chen, John Rowe, Toni Antonucci, Lisa Berkman, Axel Börsch-Supan, Laura Carstensen, Dana Goldman, Linda Fried, Frank Furstenberg, James Jackson, Martin Kohli, Jay Olshansky, David Rehkopf, John Rother, Julie Zissimopoulos

**Affiliations:** 1 Saw Swee Hock School of Public Health, National University Health System and National University of Singapore, Singapore, Singapore; 2 Schaeffer Center for Health Policy and Economics, University of Southern California, Los Angeles, CA USA; 3 London School of Hygiene & Tropical Medicine, London, UK; 4 Mailman School of Public Health, Columbia University, New York, NY USA; 5 College of Literature, Science and the Arts, (Department of Psychology), University of Michigan, Ann Arbor, MI USA; 6 T.H. Chan School of Public Health, Harvard University, Boston, MA USA; 7 Max Planck Institute for Social Law and Social Policy, Munich, Germany; 8 Stanford Center on Longevity, Stanford University, Stanford, CA USA; 9 Sol Price School of Public Policy, University of Southern California, Los Angeles, CA USA; 10 Department of Sociology, College of Arts and Science, University of Pennsylvania, Philadelphia, PA USA; 11 Institute for Social Research, University of Michigan, Ann Arbor, MI USA; 12 European University Institute, Italy and Bremen International Graduate School of Social Sciences, Bremen, Germany; 13 School of Public Health, University of Illinois, Champaign, IL USA; 14 School of Medicine, Stanford University, Stanford, CA USA; 15 National Coalition on Health Care, Washington, DC USA

**Keywords:** Scientific community, Social sciences, Ageing

## Abstract

We have previously presented a multidimensional Aging Society Index, a weighted summation of five domains central to successful adaptation to societal aging: well-being, productivity and engagement, equity, cohesion and security, as a tool to assess countries’ adaptation to demographic transformation. As the index was based on data from developed countries and some of the individual metrics or weightings may not be well suited for application to low- and middle-income countries, we here present the scores on a modified index (Global Aging Society Index) on 143 countries distributed across the span of economic development. Only 5 out of 143 (3.5%) countries had higher scores for women than men. Countries with the most notable gender differences were primarily low-income countries. The multidimensional index permits cross-national comparisons and may facilitate the identification of targets for developing policies and programs to enhance the likelihood that older persons will age successfully.

## Abstract (web summary)

The authors score and rank the adaptation to societal aging across 143 countries using a newly developed index called the Global Aging Society Index, which may be helpful to identify targets for the development of policies and programs to enhance the likelihood that older people will age successfully.

## Main

Societal aging is a success story. It is closely tied to public health and medical achievements in reducing the risk of premature death and controlling or preventing disease. It is linked in a virtuous cycle to social and economic development^1^. These relationships, combined with progressive reductions in fertility rates, have resulted in almost all countries experiencing dramatic growth in the size and proportion of their older populations. The number of people over 65 years worldwide, which was 703 million in 2019, is projected to double to 1.5 billion by 2050 (ref. ^2^). As countries’ populations continue to age, these demographic trends will likely have substantial societal impacts, and societal responses must be evidence-informed if social and economic development is to continue^2^.

In high-income countries, the likelihood of a newborn living to age 90 has risen from 4.8% in 1950 to 26.7% today and is projected to be 50% by 2060 (ref. ^3^). These demographic changes drive modifications in family structures, with fewer young and middle-aged individuals responsible for supporting older family members. In many countries, these family support changes, when coupled with poor social insurance programs, low pension wealth, low savings rates and long lives, especially in women, place a large portion of the older population at risk of poverty. To adapt to the challenges associated with societal aging, countries must develop policies and programs that will essentially re-engineer many of their core institutions.

Previous approaches to assessing societal aging^4–6^, including recent publications from our group^7,8^, have generally focused on the Organisation for Economic Co-operation and Development (OECD) countries, where most members are high-income economies with a high Human Development Index; however, it is estimated that about 80% of adults aged 60 and above will reside in low- and middle-income countries by 2050 (ref. ^9^) and the effects of population aging will be more notable in these countries as existing services and infrastructure are less prepared for an aging society^10^.

It takes a substantial amount of time for appropriate policies to be implemented, such as building a support structure for long-term care and training geriatric doctors and allied workers. Future planning is also required to build infrastructure to cater to the needs of older adults. In South Korea, for example, public transport has always been relatively inaccessible for people who are physically disabled, with a lot of stairs in underground train stations; however, the Korean government is starting to respond to the needs of older adults and disabled people by installing lifts in all metro stations, with currently 93% of metro stations in Seoul having lifts installed^11^. In many countries, shops and eateries have stairways and steps, limiting access for people with physical disabilities^12^. Retrofitting to overcome these sorts of challenges is expensive. Lower-income countries can learn from the mistakes of more developed settings to ensure development occurs in a way that will prepare the country for the population aging that will inevitably come.

Several indices have been developed to assess country preparedness for population aging, including the Global AgeWatch Index 2015 (ref. ^4^), the EU Active Aging Index 2018 (ref. ^13^) and the Human Development Index 2020 (ref. ^14^); however, even though these are useful in determining how effectively countries deal with population aging, they have limitations in comprehensively assessing it. The Human Development Index only includes four items: life expectancy, national GDP, and two measures of educational attainment, and it is not tailored specifically for older adults; the Active Aging Index has heavy weightage (70%) on productivity and engagement domains, and the Global AgeWatch Index lacks measures in cohesion as well as volunteering and retraining, which are crucial components of successful aging.

**Fig. 1:**
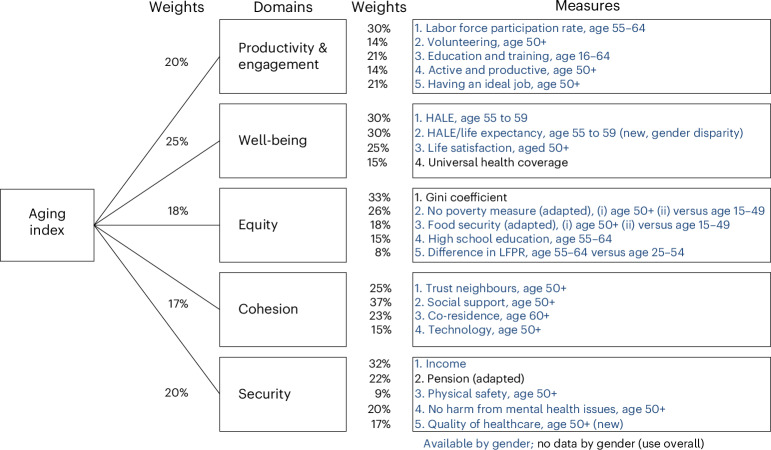
Global measures in the Aging Index. Measures and weights categorised by domains.

To better gauge societal preparedness for this substantial demographic transformation, we have previously undertaken work to explore how high-income countries are adapting to population aging^7,8^. Inspired by the Successful Aging paradigm of Rowe and Kahn and informed by extensive discussions within the Aging Society Research Network, our original work considered adaptation through five key domains: Productivity and Engagement, Well-being, Equity, Cohesion and Security^15^. For this study, we refined this index (Fig. 1) through further consultation with an expert panel experienced in low- and middle-income country (LMIC) issues and expanded the data sources to allow the assessment of 143 low-, mid- and high-income countries. We aimed to enable countries to benchmark their performance against countries at a similar economic development stage, thus stimulating appropriate policy and program responses.

## Results

### Overall world rankings

**Fig. 2:**
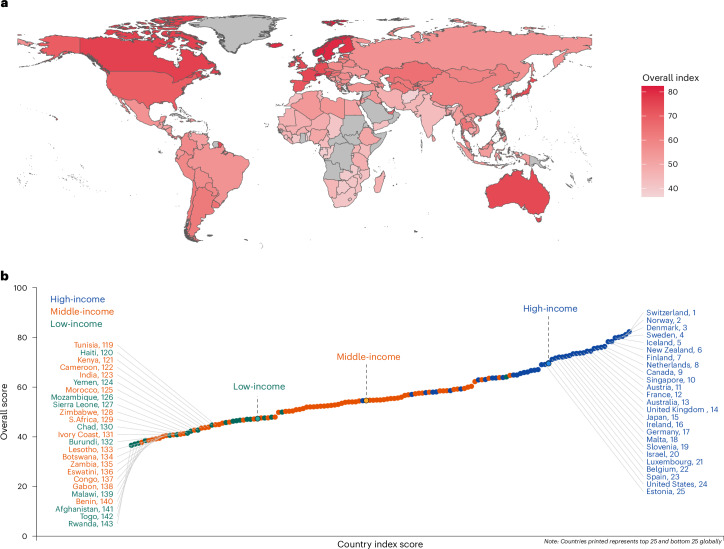
Global Aging Society Index and world rankings. **a**, Aging Society Index world map. **b**, Overall ranking.

High-income countries dominated the top rankings (Fig. 2a,b), with a mean score of 24.9 (95% CI 21.3–28.6) higher than low-income countries; however, after adjusting for the macro variables, the mean score between high and low-income countries was not statistically significant (*β* = 1.34, 95% CI −5.06–7.75) (Supplementary Table 3). The overall Global Aging Society Index ranged from 36.6 for Rwanda in Africa to 82.3 for Switzerland in Europe. Most of the top ten performing countries were from Europe (Supplementary Fig. 7). There are some differences in country rankings compared to the ones in the previous work^7^. For instance, Switzerland is now at the top of the chart compared to Norway; however, Switzerland was not part of our earlier analysis, whereas Norway, which had previously ranked first, is currently placed second. Denmark and Finland climbed the rankings and scored higher than Ireland, Japan and the United States. This is due to the difference in measures such as having an ideal job, where Denmark was 5th, Finland 11th, Ireland 17th, United States 43rd and Japan 70th. Another of these measures includes the proportion of the population aged 50+ years with access to the internet, where Denmark was 2nd, Finland 5th, Ireland 20th, United States 8th and Japan 41st. These have resulted in countries ranking differently from the previous study^7^.

### Domain world rankings

**Fig. 3:**
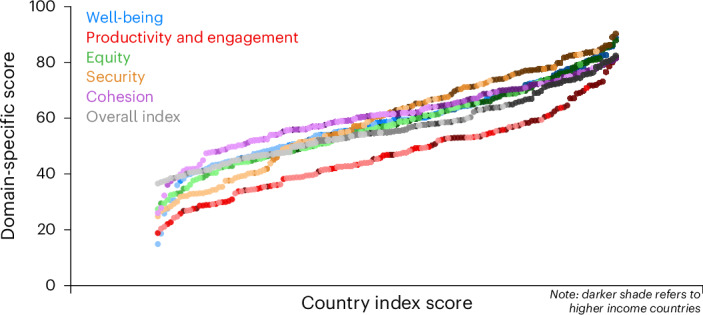
Domain world rankings, overall. Countries’ rankings for domains and overall index.

Countries scored lowest in the productivity and engagement domain (mean = 46.8, s.d. = 14.1, 95% CI 44.5–49.1), followed by the equity domain (mean = 57.5, s.d. = 14.3, 95% CI 55.1–59.8), well-being domain (mean = 58.4, s.d. = 14.2, 95% CI 56.0–60.7) and security domain (mean = 59.3, s.d. = 17.5, 95% CI 56.4–62.2). Countries scored the highest for cohesion (mean = 60.6, s.d. = 11.6, 95% CI 58.7–62.5). The domain scores ranged from 14.9 (Afghanistan) to 88.8 (Singapore) for well-being (Supplementary Fig. 2e), 18.8 (Jordan) to 81.7 (Switzerland) for productivity and engagement (Supplementary Fig. 3f), 27.4 (Eswatini) to 87.8 (Norway) for equity (Supplementary Fig. 4h), 26.0 (Benin) to 81.3 (Bahrain) for cohesion (Supplementary Fig. 5e) and 24.8 (Liberia) to 90.2 (Norway) for security (Supplementary Fig. 6f) (Fig. 3). The coefficient of determination between domains was mainly low or moderate (0.07 < *r*^2^ < 0.59). As observed in our previous work, this lack of strong associations between performance in various domains underscores the need for a multidimensional index. A country’s GDP level was significantly associated with the aging index scores in most of the domains.

### Well-being

High-income countries top the chart for the well-being domain (Supplementary Fig. 2e) with longer healthy life expectancy (Supplementary Fig. 2a), higher life satisfaction and better universal health coverage (Supplementary Fig. 2c,d). The mean well-being score in high-income countries was 28.2 (95% CI 23.0–33.4), higher than in low-income countries (Supplementary Table 3); however, high-income countries also spend a large proportion of their life in poor health (Supplementary Fig. 2b).

### Productivity and engagement

The top ten countries of the productivity and engagement domain were primarily high-income countries (Supplementary Fig. 3f). The mean productivity score in high-income countries was 15.3 (95% CI 9.21–21.4) higher; however, after adjusting for macro variables, the mean was 15.8 (95% CI 3.28–28.4) higher (Supplementary Table 3). Measures of this domain were weighted heavily on the country’s labor force participation rate (30%), where low and middle-income countries scored well (Supplementary Fig. 3a); however, high-income countries provide more retraining opportunities (Supplementary Fig. 3b). Indonesia was at the top for volunteering (Supplementary Fig. 3c).

### Equity

Most high-income countries were more equitable (Supplementary Fig. 4h), with a mean equity score of 21.9 (95% CI 16.2–27.6) higher than low-income countries; however, after adjusting for macro variables, the mean difference was no longer statistically significant (*β* = 0.55, 95% CI −11.1–12.2) (Supplementary Table 3). The Gini coefficient measures the degree of inequality in the distribution of income, where European countries had the lowest inequality and African countries had the highest inequality (Supplementary Fig. 4a). In addition, older adults in high- and middle-income countries were more likely to live comfortably, have food security (Supplementary Fig. 4b,d) and have attained at least a high school education (Supplementary Fig. 4f). In terms of differences between older adults (age 50 and above) and the working population (age 15–49), Asian countries perform well in no poverty risk (Supplementary Fig. 4c) and in food security (Supplementary Fig. 4e). LMIC countries top the charts for differences in labor force participation rate (Supplementary Fig. 4g).

### Cohesion

Most high-income countries have a higher cohesion (Supplementary Fig. 5e), where the mean cohesion score was 17.1 (95% CI 11.9–22.3) higher than in low-income countries; however, after adjusting for macro variables, the mean difference was no longer statistically significant (*β* = −5.93, 95% CI −16–4.15) (Supplementary Table 3). The top countries in cohesion were mainly from Asia and Europe (Supplementary Fig. 5e). Countries with the highest proportion of older adults who trust their neighbors (Supplementary Fig. 5a) and have strong social support (Supplementary Fig. 5b) were from Asia and Europe, whereas co-residence was highest in Asia and Africa (Supplementary Fig. 5c).

### Security

High-income countries top the chart for security, where seven out of the top ten countries were from Europe (Supplementary Fig. 6f). The mean security score in high-income countries was 40.0 (95% CI 33.8–46.2) higher than in low-income countries; however, after adjusting for macro variables, the mean was no longer statistically significant (*β* = −2.36, 95% CI −13–8.31) (Supplementary Table 3). European countries perform well in income (Supplementary Fig. 6a) and pension (Supplementary Fig. 6b). In contrast, Asian countries feel the safest walking alone at night (Supplementary Fig. 6c), have the least harm due to mental health issues (Supplementary Fig. 6d) and are most satisfied with the quality of healthcare (Supplementary Fig. 6e).

### Gender differences

**Fig. 4:**
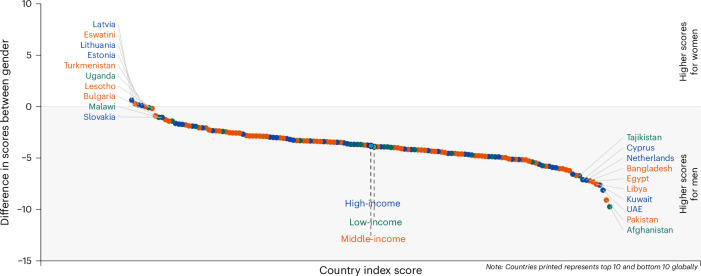
Global Aging Society Index difference, women – men. Countries on the left of the ranking have higher scores for women and countries on the right have higher scores for men.

**Fig. 5:**
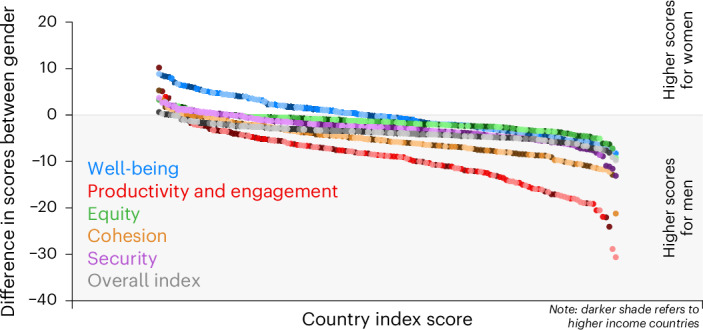
Domain scores differences, women – men. Countries on the left of the ranking have higher scores for women and countries on the right have higher scores for men.

We computed the differences between women and men, where a positive difference implies higher scores for women. Only five countries had higher scores for women than men (Fig. 4). The mean overall index score for females was 3.85 (95% CI 1.23–6.47) (Supplementary Table 3) lower than males. Countries with the largest gender differences mainly were low-income countries, where males had an average score of 3.95 (95% CI 0.2–7.69; *P* = 0.04) more than females among lower-income countries. By domains, the range of gender differences was largest for the productivity and engagement domain, from −30 in Bangladesh to 10.2 in Finland (Fig. 5). In most countries, men performed better in equity (mean = 1.69, 95% CI −1.58–4.97), cohesion (mean = 5.43, 95% CI 2.71–8.15), and security (mean = 2.99, 95% CI −1.03–7) domains (Fig. 5 and Supplementary Table 3). A higher score in some domains for one gender may not necessarily reflect a more advantageous position. For example, the higher score for men in productivity could reflect lower economic security driving individuals to remain in the workforce.

### Sensitivity analysis

**Extended Data Fig. 1:**
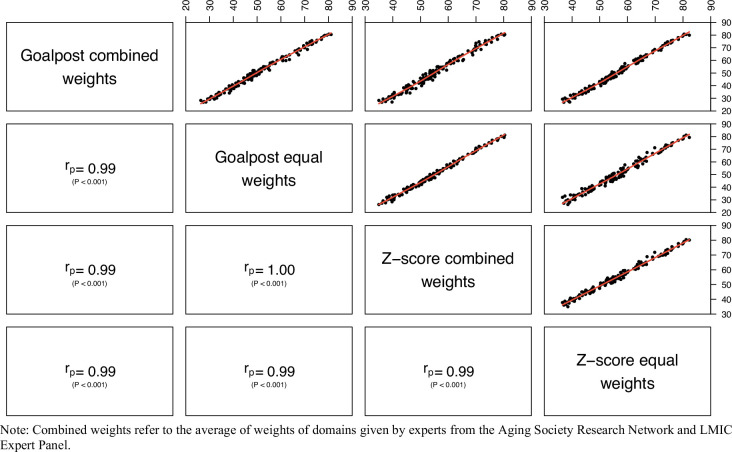
Correlation (Pearson correlation test) between scoring methods and weighting schemes for overall Aging Society Index scores (Overall). Correlation (Pearson correlation test) between scoring methods and weighting schemes for overall Aging Society Index scores for all genders. Note: Combined weights refer to the average of weights of domains given by experts from the Aging Society Research Network and LMIC Expert Panel.

**Extended Data Fig. 2:**
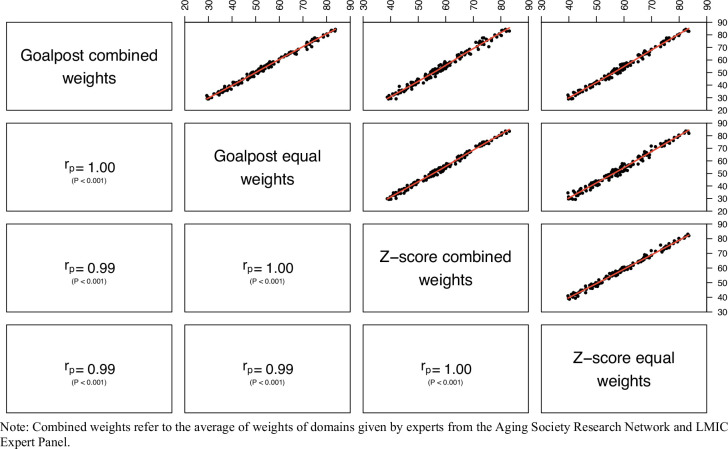
Correlation (Pearson correlation test) between scoring methods and weighting schemes for overall Aging Society Index scores (Male). Correlation (Pearson correlation test) between scoring methods and weighting schemes for overall Aging Society Index scores for males. Note: Combined weights refer to the average of weights of domains given by experts from the Aging Society Research Network and LMIC Expert Panel.

**Extended Data Fig. 3:**
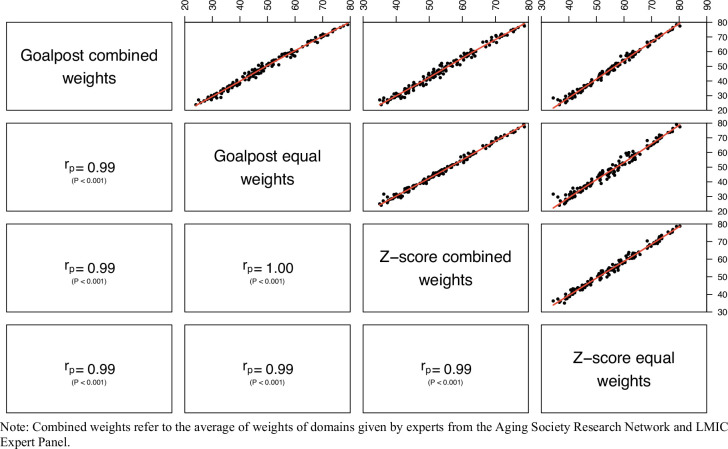
Correlation (Pearson correlation test) between scoring methods and weighting schemes for overall Aging Society Index scores (Female). Correlation (Pearson correlation test) between scoring methods and weighting schemes for overall Aging Society Index scores for females. Note: Combined weights refer to the average of weights of domains given by experts from the Aging Society Research Network and LMIC Expert Panel.

Sensitivity analysis is presented in Extended Data Figs. 1–3. Our results were robust in terms of scoring methods and weighting schemes. Furthermore, comparing methods and weights yielded a high correlation (*r* ≥ 0.9).

## Discussion

**Extended Data Fig. 4:**
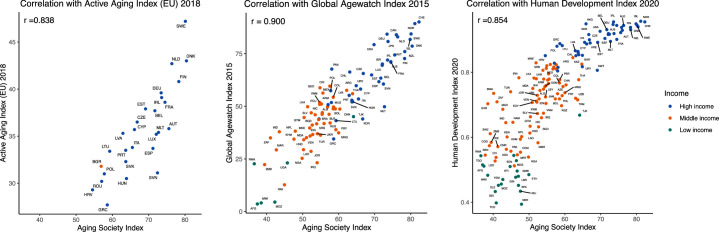
Correlation between Global aging society index and other indices. Correlation between Global aging society index and Active aging index, Global agewatch index and human development index.

We used survey data from the Gallup World Poll and six open sources, including the World Bank, the United Nations and the Global Burden of Disease, to develop an index that estimates countries’ adaptation to societal aging. To our knowledge, this is the first study to evaluate societal aging on such an extensive scale. Overall, low- and middle-income countries score lower in adapting to societal aging, and our results highlight the potential gaps and the gains that would be possible with effort in these countries. Correlation plots between the Global Aging Society Index and other existing indices are presented in Extended Data Fig. 4. The respective correlation coefficients lie between 0.84 and 0.90, which indicates a relatively strong linear relationship between the Global Aging Society Index and these other indices; however, the Global Aging Society Index includes five domains from older adults’ experiences in low-, mid- and high-income settings. In addition, the index also investigates a substantially larger number of countries (*n* = 143) than the Active Aging Index (*n* = 28) and the Global AgeWatch Index (*n* = 96) (Supplementary Table 4). The index that was most inclusive in capturing 189 countries, the Human Development Index (HDI), only includes four items: life expectancy, national GDP and two measures of educational attainment, and these measures are not specific for older adults. Thus, the HDI provides a much less robust assessment of societal adaptation than our index of five major domains based on over 20 measures. A comparison of the country-specific assessments of our index and the HDI is also included in Supplementary Table 5.

The top performance of Switzerland in the overall aging index reflects its strong performance in security (rank second), well-being (third) and productivity and engagement (first). It has often been cited as one of the best places to grow old^16^. Older adults in Switzerland have high life satisfaction (joint first) and feel safe walking alone at night (second in Europe). We also found that 94% of older adults are satisfied with the quality of healthcare (joint first)^17,18^. Policies for Switzerland’s aging society show that the Swiss government is working on improving the pension system, integrating the health system better and controlling healthcare costs despite its good outcomes currently^19^. Yet, even for Switzerland, there are areas for improvement, such as labor force participation rate and volunteering, as other countries perform better in these measures.

For the well-being domain, Singapore ranks top with the world’s longest healthy life expectancy at older ages, strong universal health coverage (joint first in Asia), high life satisfaction (fifth in Asia) and a high proportion of life expectancy spent in good health (seventh in Asia). Despite a total health expenditure of 4.08% (ref. ^20^) of the gross domestic product in 2019, compared to 12.5% (ref. ^20^) for high-income countries, Singapore has achieved good health outcomes. In promoting long-term, transformational change, Singapore’s Ministry of Health has implemented a set of health transformation efforts, including preventive efforts such as screening, immunization, health promotion (such as the National Steps Challenge and Healthier Dining program) and education, where spending doubled from SGD 723 million in 2014 to SGD 1.47 billion in 2017 (ref. ^21^). In addition, Singapore is also developing the first-of-its-kind ‘Health District @ Queenstown’, with backing from multi-stakeholders to drive efforts in the built environment, preventive health and care delivery programs, social science research and technology partnerships with government, academia and industry. Successful initiatives from the health district will be included in future rejuvenation plans and scaled to other estates^22^. Singapore has also recently intensified its efforts in chronic disease prevention and management through the implementation of Healthier SG from 2023, a nationwide program led by the Singapore Ministry of Health. This initiative aims to transition the healthcare system from a reactive treatment model to one focused on proactive preventive care. Under the program, residents are encouraged to take greater ownership of their health by enrolling with a family doctor, who will collaborate with them to develop a personalized health plan tailored to their specific needs. The Singapore government has allocated SGD 1 billion over the next 3 years for the implementation of Healthier SG, with an anticipated annual operating cost of SGD 400 million thereafter^23^.

We also find that low- and middle-income countries tend to do poorly, especially in terms of well-being and security, resulting in low scores in the overall aging index. For example, Kenya is ranked 128th in well-being, 131st in equity, 132nd in cohesion and 120th in security, with Mozambique ranked 133rd in well-being, 141st in equity, 120th in cohesion and 118th in security. Many of these countries are relatively young; however, some are expected to experience rapid population aging in the future^2,24^.

In these settings, older individuals with lower labor income face substantial challenges with healthcare^25^. In the long run, if the health and social security system in these countries remains inadequate to meet older adults’ needs, a substantial financial burden will fall on the individuals or their families, which is likely to have broader economic consequences^24^. The Commission for a Global Roadmap for Healthy Longevity^26^ proposes that addressing the needs of older adults will require countries to shift their healthcare approach toward integrated person-centered care instead of the usual disease-centered care.

Average life expectancy has dramatically increased during the 20th century across most countries, with infectious or acute diseases waning to chronic diseases^27^. Many countries are experiencing a rise in chronic diseases^27^, with some having implemented or starting to implement policies to curb chronic diseases. For instance, Australia has implemented the National Strategic Framework for Chronic Conditions and the Implementation Plan for the National Aboriginal and Torres Strait Islander Health Plan to aid in minimizing and controlling the prevalence of various chronic conditions^28^. Our study found that although Japan ranked one of the top countries for healthy life expectancy, it ranked 38th regarding the proportion of life spent in good health. The Japanese are spending an average of 5.6 years in poor health, which is longer compared to other countries in low- (mean = 3.7 years, 95% CI 3.5–3.8), middle- (mean = 4.4 years, 95% CI 4.2–4.6) and high-income countries (mean = 5.2 years, 95% CI 5.0–5.3). There has been a debate about whether the gains in life expectancy are spent in good or poor health. Forty years ago, Fries introduced the idea of ‘compression of morbidity’^29^, where increased life expectancy is accompanied by shortening the length of morbid life, lower incidence of diseases and higher age-onset^30,31^; however, recent findings suggest otherwise^32–34^.

Next, we compared average-performing countries in the aging index by income categories. Spain was ranked 23rd out of 46 high-income countries, Indonesia was ranked 38th out of 77 middle-income countries and Haiti was ranked 11th out of 20 low-income countries.

Despite being an average-performing high-income country, Spain ranks third in social support and joint first in pension. Spain provides one of the highest unemployment benefits in the world^35^ for those who have worked in the last 6 years. There is also an Active Placement Income benefit for unemployed persons over 52 (ref. ^36^). This likely influenced the low labor force participation rate, where Spain ranked 30th out of the 46 high-income countries. The high unemployment rate is twice that of the EU8 countries^37^. Also, most job offers are temporary contracts and 90% of these temporary jobs do not convert to permanent jobs^37^. These reasons suggest why Spain is ranked extremely low in labor force participation. Spain also ranks 8th for Healthy Life Expectancy (HALE) and joint 13th for Universal Health Coverage.

Our results show that nine of the bottom ten countries are based in Africa (Supplementary Fig. 7). Most of the older people in these countries have been reported not to have sufficient access to affordable healthcare^38^. The majority also did not have evidence of including non-communicable disease treatments in their public healthcare, and hardly any included mental health in their healthcare policies. Healthcare spending in Rwanda^39^ (last overall) consists of only 6.88% of the total GDP and out-of-pocket spending consists of about 6.44% of total healthcare expenditure.

Gender inequality has been highlighted as an important global health issue^40^. We found that in almost all countries, women only have higher scores in the well-being domain, with men having higher scores in the other domains, leading to the overall index favoring men. This finding is similar to our earlier study^8^. At the societal level, such inequality in the social determinants of health limits the potential of societies to secure societal well-being and make cities safe, inclusive and sustainable^41^. As such, the Sustainable Development Goals hope to achieve gender equality and empower all women by valuing unpaid care and domestic work, ensuring women’s full and effective participation in political, economic and public life, and providing universal access to health and rights^41^. In addition, the World Health Organization and United Nations (UN) member states came up with a 10-year global plan of action in 2020, with the goal to ensure that all older people can live long and healthy lives. This is formally known as the UN Decade of Healthy Aging (2021–2030)^42–44^. As such, our index allows countries to measure their progress and areas for improvement to help older adults age successfully.

Our study has some limitations. First, our global cross-sectional comparison does not identify trends over time, which would only be possible with repeated panel data. Some measures were not gender-disaggregated in some domains (Fig. 1). Also, we could only include measures that are available for all countries. Thus, we could not include rigorous mental health measures. For Gallup measures, gender data for older adults were not directly available (Supplementary Table 1). Nevertheless, sensitivity analysis comparing rescaled data and the overall rates yields similar results with high correlation (*r* ≥ 0.96). Next, we used measures from older adults wherever possible, but some were not age-stratified. For LMIC countries with much lower life expectancy, there may be an issue of survival bias. As such, we have lowered the age from 65+ to 50+ where possible. Finally, other experts might derive the measures and domain weights differently. We included a comparison using equal weights, and our results were robust regarding computation methods and weighting strategies (Supplementary Fig. 8).

An effective response to population aging can benefit countries in numerous ways. Not only can they reduce healthcare costs, but countries can also tap into the potential of healthy older people, using their experience and wisdom, which will greatly benefit societies globally in the long run. LMICs generally score lower in all domains and they can learn from the experience of those high-income countries scoring higher; however, the disparity within each income group shows that LMICs can learn from the mistakes and successes of higher-income settings. While they may still be early in the demographic transition, now is the time for these countries to lay sustainable foundations and invest while the absolute number of older adults is small. We hope that by allowing a nuanced comparison of the experience of countries in all settings, our index can help prioritize action for countries at all levels of development.

## Methods

Our research study complies with all relevant ethical regulations and is approved by the National University of Singapore Institutional Review Board.

### Data

Data for the aging index measures were taken from the Gallup World Poll and other open sources, including (1) the International Labor Organisation database; (2) World Bank Open Data; (3) the Global Sustainable Development Goals Indicator Database; (4) Global Burden of Disease Results Tool; (5) UN Data; and (6) OECD data. The latest available cross-sectional country and gender-specific data were obtained at a single time point between 2015 and 2019. Data beyond 2019 were excluded due to the potential impact of COVID-19 on measures. Wherever possible, gender-specific data were also obtained (Supplementary Table 1). Variables that were considered potential confounders used in regression analysis were obtained at a single time point in 2019 and were obtained from World Bank Open Data.

We took the list of 148 countries from the Gallup World Poll country set in 2018 and 2019 (ref. ^45^) as it was the most frequently used source to obtain data for variables. We removed five countries, namely Ghana, Kosovo, Northern Cyprus, Saudi Arabia and Taiwan, due to a lack of data on variables from other sources. As a result, our study included 143 countries with the most available data, representing 95.4% of the world’s population. Regions in this study were defined using UN regions and incomes were categorized using the World Bank’s income definitions. Out of the 143 countries, most countries (*n* = 133) have fewer than 20% missing variables. Of the 25 measures, most measures (*n* = 20) have fewer than 10% of missing countries (Supplementary Table 7). For countries that had missing data for the measure(s), we imputed these measures using region- and income-specific averages.

The adapted measures use available global data and retain consistency in the measures. Our final index constitutes the exact five domains (Fig. 1) included in our initial work on the OECD and builds the domain scores based on 25 measures instead of the original 19 measures. As our initial work did not specifically account for issues that might be particularly important in LMICs, we assembled an LMIC expert panel comprising 11 scholars with substantial experience relevant to aging and low or middle- income settings. The panel spanned multiple disciplines, including demographers, sociologists, economists, psychologists and policy experts. Their details are available in Supplementary Table 2a. We familiarized this expert panel with our methodology, and the LMIC expert panel reviewed each metric in each domain in the original index to assess its relevance to LMIC. Many changes were made, including adding, deleting and modifying measures, and the finalized metrics were adjusted based on data availability. The panel members then individually provided weights for the individual metrics and the domains adapting from the Delphi method, and the weights established were added to the revised index, which we refer to as the Global Aging Society Index. The total weights established by both the experts from the Aging Society Research Network and the LMIC expert panel are included in Supplementary Table 2b. In addition to these changes referred to above, as the life expectancy of low-income countries is lower than in high-income countries^46^, we lowered the age of older persons from 65 and above to 50 and above for all measures where applicable.

A full description of the Aging Society Index has been detailed in previous papers^7,8^ and will only be summarized here. The domains that characterize successful aging in society are defined as follows:

#### Well-being

A successfully aging society provides healthcare informed by a sophisticated understanding of the healthcare needs of older persons^47,48^. Previously, the Well-being domain was attributed to an objective measure, HALE at age 65 (70%), and a subjective measure, life satisfaction at age 50 (30%). In the subsequent gender differences work^8^, to correct for uniformly longer life in women than men, we modified the HALE measure by normalizing it with life expectancy (LE) at age 65 to represent the proportion of disease-free LE at age 65 by taking a ratio of HALE and LE (HALE:LE). Continuing our previous published works, this study used HALE (30%) and a ratio of HALE and LE (30%) as objective measures. This was intended to measure both HALE and the proportion of disease-free LE at age 55. We also kept the self-reported life satisfaction at age 50 (25%). In addition, our study also included a new measure on healthcare coverage in a country (15%). The proportion of universal health coverage represents the extent of health services the population receives. Countries will need to factor in the increasing healthcare needs of the growing adult population to aim toward attaining universal health coverage^49^. Our previous study dropped this measure due to a lack of variability among OECD countries (most had nearly 100% universal health coverage).

#### Productivity and engagement

A successfully aging society facilitates the engagement of older persons in society through work for pay or volunteering^50,51^. The measures were labor force participation rate (30%), volunteering (14%) and retraining (21%). The effective retirement age was removed due to the high collinearity with labor force participation rate and the LMIC expect panel suggested the inclusion of variables (1) feeling active and productive (14%) and (2) whether they felt that their current job is ideal (21%).

#### Equity

A successfully aging society ensures equitable distribution of resources equitably across generations, lessening the gap between the ‘haves’ and the ‘have nots’^52,53^. The measures were the Gini coefficient (33%), food security (9%), no poverty measure (13%) and the percentage of older adults’ with high school education (15%). The LMIC expert panel also suggested adding differences between older adults (age 50 and above) and the working population (age 15 to 49) in food security (9%), no poverty risk (13%) and labor force participant rate (8%).

#### Cohesion

A successfully aging society maintains social connectedness within and between generations^54,55^. The measures were individuals’ trust in family, friends, and/or neighbors (25%), as well as their social support (37%) and co-residence (23%). The LMIC expert panel suggested the addition of access to technology (15%).

#### Security

A successfully aging society provides economic and physical security for older persons^56^. This domain measures safety and support for retirement, including income (32%), pension (22%), physical safety (9%) and satisfaction with the quality of healthcare (17%), which is used as an alternative to public expenditure on long-term care as there is a lack of global data available for the latter variable. The LMIC expert panel suggested the addition of no harm due to mental health issues (20%).

### Statistics and reproducibility

We constructed the measures overall and by gender. Detailed methodology for the construction has been published previously^7,8^. All measures used in our study were converted into positive indicators, where a higher value represents a better outcome. Each country’s score was standardized by the minimum and maximum values for each measure and was calculated using the goalpost method^7,8^:

$$\mathrm {{Goalpost}}=\frac{{\mathrm {actual}}-\min }{\max -\min }\times 100$$

For the population-level analysis, a score of 0 and 100 was given to the country with the lowest and highest values for each variable, respectively. By gender, a score of 0 and 100 was given to the men or women in the country with the lowest and highest values for each variable, respectively.

Each domain score was calculated as a weighted summation of the scores of the measures within the domain. The overall composite Aging Society Index was then calculated as a weighted summation of the domain scores (Fig. 1). These weights (Supplementary Table 2b) were established previously by a 14-member interdisciplinary group of experts from the Aging Society Research Network^7^ and the new 11-member LMIC expert panel (Supplementary Table 2a). The weights of measures within all domains have been modified to accommodate the additional measures.

Sensitivity analysis was conducted using *z*-scores and equal weights. We calculated the *z*-scores for each measure and used equal weights for all domains and measures. The robustness of our results was assessed using Pearson’s correlation coefficient between the overall Aging Society Index score from the goalpost method and *z*-scores with network and equal weights, respectively.

We calculated the difference in scores within a country (women − men). A *t*-test was performed on the difference, and statistical significance was measured at a 5% level. Spearman and Kendall’s correlations were used on domain scores and measures within each domain, respectively, where the latter method accounted for tied observations within the measure.

We used nested linear regression to estimate the association between the countries’ scores (overall index score and by domain) with variables that are not included in the index calculation, potentially associated with the outcome of the aging index score. Model M1 included the income category, whereas M2 adjusted for log GDP per capita (adjusted for purchasing power parity), M3 further adjusted for the proportion of older adults aged 65+ in the population and M4 was the fully adjusted macros variables model, where the proportion of seats held by women in national parliaments was included. For gender differences regression, we used gender-specific scores (overall index score and by domain), adjusting for gender differences in the percentage of older adults in the population (Supplementary Table 3). We hypothesized that higher GDP generally results in an improvement in various measures, such as higher healthcare resources^57^ and higher life satisfaction^58^, thus improving well-being. We expected it also to improve food security^59^ and poverty risk, improving equity. We also hypothesized that with a higher percentage of older adults in the population, more age-focused policies would be implemented to enhance societal aging. Also, we hypothesized that the countries with a higher percentage of women in parliament would have policies that improve cohesion and improve societal aging. We also included the 95% confidence interval above and below the regression coefficients by adding or subtracting 1.96 × standard error of the regression coefficients estimates, respectively.

Data analysis was performed using R (v.4.1.3). Results of measurement scores can be found in Supplementary Figs. 2–6.

### Reporting summary

Further information on research design is available in the Nature Portfolio Reporting Summary linked to this article.

## Supplementary information

Supplementary Information Supplementary Figs. 1–7 and Tables 1–7.

Reporting Summary

## Data Availability

All statistical data associated with this study are in the main text or supplementary material. Data taken from open sources, which include (1) the International Labor Organisation database (https://ilostat.ilo.org); (2) World Bank Open Data (https://data.worldbank.org); (3) The Global Sustainable Development Goals Indicator Database (https://unstats.un.org/sdgs/dataportal); (4) Global Burden of Disease Results Tool (https://vizhub.healthdata.org/gbd-results/); (5) UN Data (https://population.un.org/LivingArrangements/index.html#!/countries/484); and (6) OECD data (https://data-explorer.oecd.org), are publicly available. A complete list of variables and their respective sources are listed in Supplementary Table 1. Data from the Gallup World Poll are not publicly available; however, they are available from the corresponding author upon reasonable request.
