# Supplementary material for: A global analysis of adaptation to societal aging across low-, middle- and high-income countries using the Global Aging Society Index
Source: Nat Aging. 2024 Dec 27;5(1):113–21. doi: 10.1038/s43587-024-00772-3 (PMC11754087; doi:10.1038/s43587-024-00772-3)

nature aging

Supplementary information https://doi.org/10.1038/s43587-024-00772-3

A global analysis of adaptation to societal aging across low-, middle- and high-income countries using the Global Aging Society Index

In the format provided by the authors and unedited

Supplementary table S1. Description and sources for measures used in aging index

Domain (network weights/ equal weights)	Measure	Description	Gender	Weights (network/ equal)	Source[1]	Download date	Data years
Productivity and engagement (20.4%/20%)	Labour force participation rate, age 55-64	The proportion of population age 55-64 in the labour force	Yes	30%/20%	International Labour Organisation (ILO) Database	16-Apr-24	2018
	Volunteering, age 50+	Proportion of population aged 50+ volunteering their time to an organisation in the last month	Yes, weighted	14%/20%	Gallup World Poll	14-May-21	2018-2019
	Education and training, age 16-64	Participation rate of youth and adults (aged 16 to 64) in formal and non-formal education and training in the previous 12 months	Yes, only 97 countries	21%/20%	Sustainable Development Goals (SDG) Indicator Database	8-Mar-21	2001-2018
	Felt active and productive, 50+	Proportion of population aged 50+ who felt active and productive everyday in the last seven days.	Yes, weighted	14%/20%	Gallup World Poll	15-Apr-24	2013-2015
	Having an ideal job, 50+	Proportion of employed population aged 50+ who felt that their current job is ideal for them.	Yes, weighted	21%/20%	Gallup World Poll	15-Apr-24	2010-2013
Well-being (24.5%/20%)	Healthy life expectancy HALE), age 55-59	Healthy life expectancy at age 55 to 59	Yes	30%/25%	Global Burden of Disease Results Tool	29-Apr-24	2019
	Ratio of healthy life expectancy and life expectancy at age 55-59	Healthy active life expectancy normalized by life expectancy, at age 55	Yes	30%/25%	Global Burden of Disease Results Tool	29-Apr-24	2019

	Life satisfaction, age 50+	Subjective well-being for population aged 50+ measured by Cantril Self-Anchoring Striving Scale	Yes, weighted	25%/25%	Gallup World Poll	14-May-21	2018-2019
	Universal Health Coverage	Coverage of essential health services for general population	No	15%/25%	Sustainable Development Goals (SDG) Indicator Database	8-Mar-21	2017
**Equity (17.9%/20%)**	Gini coefficient	Gini coefficient in general for population	No	33%/20%	World Bank	2-Mar-21	1998-2018
	No poverty risk, age 50+	Proportion of population aged 50+ living comfortably or getting by on current income	Yes, weighted	13%/10%	Gallup World Poll	14-May-21	2018-2019
	Difference in no poverty risk	Difference in proportion of population aged 50+ living comfortably or getting by on current income, relative to proportion of population aged 15-49	Yes, weighted	13%/10%	Gallup World Poll	14-May-21	2018-2019
	Food security, age 50+	Proportion of population aged 50+ with enough money for food	Yes, weighted	9%/10%	Gallup World Poll	14-May-21	2018-2019
	Difference in food security	Difference in proportion of population aged 50+ with enough money for food, relative to proportion of population aged 15-49	Yes, weighted	9%/10%	Gallup World Poll	14-May-21	2018-2019
	High school attainment, age 55-64	Proportion of the population aged 55 to 64 that has attained high school or higher education	Yes	15%/20%	United Nations (UN) Data	2-Mar-21	1995-2019
	Difference of LFPR	Ratio of employment for people aged 25-54, relative to people 55-64	Yes	8%/20%	International Labour Organisation (ILO) Database	16-Apr-24	2018
**Cohesion (17%/20%)**	Trust neighbour, age 50+	Proportion of population aged 50+ who trust their neighbour	Yes, weighted	25%/25%	Gallup World Poll	14-May-21	2018

	Social support, age 50+	Proportion of population aged 50+ with friends or relatives they can count on when in trouble	Yes, weighted	37%/25%	Gallup World Poll	14-May-21	2018-2019
	Technology, age 50+	Proportion of population aged 50+ with access to internet	Yes, weighted	15%/25%	Gallup World Poll	18-Apr-24	2015-2019
	Co-residence, age 60+	Proportion of population aged 60+ not living alone	Yes	23%/25%	United Nations (UN) Data	14-May-21	1996-2019
**Security (20.2%/20%)**	Income	Average income for general population (PPP)	Yes (OECD weighted)	32%/20%	ILO and OECD	17-Jun-21	2015-2018
	Pension	Proportion of population above statutory pensionable age receiving a pension	No	22%/20%	Sustainable Development Goals (SDG) Indicator Database	8-Mar-21	2000-2019
	Physical safety, age 50+	Proportion of population aged 50+ who feel safe walking alone at night in the city they live in	Yes, weighted	9%/20%	Gallup World Poll	14-May-21	2018-2019
	No harm from mental health issues, age 50+	Proportion of population aged 50+ who do not experienced harm from mental health issues	Yes, weighted	20%/20%	Gallup World Poll	16-Apr-24	2019
	Quality healthcare, age 50+	Proportion of population aged 50+ who are satisfied with the quality of healthcare in the country	Yes, weighted	17%/20%	Gallup World Poll	14-May-21	2018-2019

[1]The Gallup world poll was one of the six data sources; the oldest age group for which data were available was 50+ years. The measures include volunteering, life satisfaction, poverty risk, food security, trust in neighbours, social support, physical safety and quality healthcare. Sex-specific data was only available for the entire population. In this case, the proportion of 50+ population responding positively to a measure was modified by the sex preferences of the entire population to derive the imputed sex preference for the 50+ population. For instance, in the case of “feeling safe walking alone at night” in Afghanistan, the proportion responding positive 50+ population was 18%. For the entire population, the proportion of men responding positively was twice that of women (16% vs. 8%). Assuming equal numbers of men and women in 50+, which was the general experience in the countries in this study, we imputed a positive response rate for 50+ for men of 24% and women of 12%. Overall and domain-specific results using this approach were very similar to results when the sex-specific data for the entire 15+ population were used to determine sex differences. Sensitivity analysis was also performed using sex-specific data of the total population aged 15+, and the results were consistent with current findings with a high correlation ($r \geq 0.96$).

Supplementary table S2A: Details of the Members of Research Network on an Aging Society and LMIC Expert Panel

Name	Location
John Rowe (Chair), MD	Columbia University Mailman School of Public Health, U.S.A
Toni Antonucci, PhD	College of Literature, Science and the Arts, (Department of Psychology), University of Michigan, U.S.A
Lisa Berkman, PhD	Harvard University T.H Chan School of Public Health, U.S.A
Axel Börsch-Supan, PhD	Max Planck Institute for Social Law and Social Policy, Germany
Laura Carstensen, PhD	Department of Psychology, Stanford University and Stanford Center on Longevity, U.S.A
Dana Goldman, PhD	USC Sol Price School of Public Policy and USC Schaeffer Center for Health Policy and Economics, U.S.A
Linda Fried, MD, MPH	Columbia University Mailman School of Public Health, U.S.A
Frank Furstenberg, PhD	Department of Sociology, College of Arts and Science, University of Pennsylvania, U.S.A
James Jackson, PhD	University of Michigan Institute for Social Research, U.S.A
Martin Kohli, PhD	European University Institute, Italy and Bremen International Graduate School of Social Sciences, Germany
Jay Olshansky, PhD	School of Public Health, University of Illinois, U.S.A and London School of Hygiene and Tropical Medicine, U.K
David Rehkopf, ScD, MPH	Department of Epidemiology and Population Health and Department of Medicine, Stanford University and Stanford Center for Population Health Sciences, U.S.A
John Rother, JD	National Coalition on Health Care, U.S.A
Julie Zissimopoulos, PhD	USC Sol Price School of Public Policy and USC Schaeffer Center for Health Policy and Economics U.S.A
Andrew Scott, PhD	London Business School, U. K
Peter Lloyd-Sherlock, PhD	School of Global Development, University of East Angina, U. K
Lowna Gie, MSc	North-West University, South Africa
Prakash Tyagi, MD	GRAVIS, India & School of Global Health and Medicine of University of Washington, U.S.A.
Jaco Hoffman, PhD	Optentia Research Unit (North-West University), South Africa
Paul K. Ayernor, PhD	African Research on Ageing (AFRAN) at the Oxford Institute of Population Ageing, U.K
John Piggott, PhD	ARC Centre of Excellence in Population Ageing Research (CEPAR), UNSW, Australia.
Tran Bich Thuy, MDM	HelpAge International, Vietnam.
Usa Khiewrord, MSc	Foundation for Older Persons’ Development (FOPDEV)
Andrew Banda, MSc	University of Zambia, Zambia
Gloria Langat, PhD	African Population and Health Research Center, Kenya

Supplementary table S2B: Relative weights of domains given by experts (%):

	Productivity Engagement	Well-being	Equity	Cohesion	Security	
**Expert1**	25	25	20	20	10	Aging Network
**Expert2**	10	40	10	10	30	Aging Network
**Expert3**	20	20	20	20	20	Aging Network
**Expert4**	40	10	20	20	10	Aging Network
**Expert5**	20	20	20	20	20	Aging Network
**Expert6**	20	30	20	10	20	Aging Network
**Expert7**	20	20	20	20	20	Aging Network
**Expert8**	10	30	20	30	10	Aging Network
**Expert9**	20	20	20	20	20	Aging Network
**Expert10**	30	25	20	15	10	Aging Network
**Expert11**	20	25	10	15	30	Aging Network
**Expert12**	20	20	20	10	30	Aging Network
**Expert13**	20	30	15	15	20	Aging Network
**Expert14**	30	28	17	14	11	Aging Network
**Expert15**	20	20	20	20	20	LMIC Expert Panel
**Expert16**	15	25	25	15	20	LMIC Expert Panel
**Expert17**	23	25	16	16	20	LMIC Expert Panel
**Expert18**	20	25	20	20	15	LMIC Expert Panel
**Expert19**	15	25	20	15	25	LMIC Expert Panel
**Expert20**	10	25	10	30	25	LMIC Expert Panel
**Expert21**	25	20	20	10	25	LMIC Expert Panel
**Expert22**	20	25	15	20	20	LMIC Expert Panel
**Expert23**	15	25	20	15	25	LMIC Expert Panel
**Expert24**	20	25	20	15	20	LMIC Expert Panel
**Expert25**	35	30	10	10	15	LMIC Expert Panel
**Average**	20.4	24.5	17.9	17	20.2	

Note: The average weights were weighted by the number of countries in each income group (high-income countries, and lower-middle income countries)

Supplementary Table S3: Regression models of overall aging index scores and domain scores adjusted for income group, gender, and macro variables.

Total:

	Overall				Productivity and Engagement				Well-being				Equity				Cohesion				Security			
VARIABLES	M1	M2	M3	M4	M1	M2	M3	M4	M1	M2	M3	M4	M1	M2	M3	M4	M1	M2	M3	M4	M1	M2	M3	M4
High Income	24.9*** (21.3 - 28.6)	1.82 (-4.63 - 8.26)	1.23 (-5.26 - 7.73)	1.34 (-5.06 - 7.75)	15.3*** (9.21 - 21.4)	13.9** (0.80 - 27.0)	15.5** (2.36 - 28.6)	15.8** (3.28 - 28.4)	28.2*** (23.0 - 33.4)	-0.60 (-10.4 - 9.19)	-2.12 (-11.9 - 7.63)	-2.05 (-11.8 - 7.71)	21.9*** (16.2 - 27.6)	2.37 (-9.28 - 14.0)	0.53 (-11.1 - 12.1)	0.55 (-11.1 - 12.2)	17.1*** (11.9 - 22.3)	-7.39 (-17.4 - 2.67)	-5.92 (-16.0 - 4.13)	-5.93 (-16.0 - 4.15)	40.0*** (33.8 - 46.2)	-0.20 (-11.1 - 10.7)	-2.45 (-13.1 - 8.23)	-2.36 (-13.0 - 8.31)
Mid Income	7.14*** (3.73 - 10.6)	-4.78** (-8.86 - -0.70)	-4.66** (-8.74 - -0.59)	-4.55** (-8.56 - -0.53)	-2.52 (-8.25 - 3.22)	-3.26 (-11.5 - 5.02)	-3.58 (-11.8 - 4.66)	-3.20 (-11.1 - 4.68)	9.41*** (4.50 - 14.3)	-5.44* (-11.6 - 0.75)	-5.14* (-11.3 - 0.98)	-5.06 (-11.2 - 1.06)	2.30 (-3.06 - 7.66)	-7.79** (-15.2 - -0.41)	-7.42** (-14.7 - -0.13)	-7.40** (-14.7 - -0.085)	5.40** (0.55 - 10.3)	-7.22** (-13.6 - -0.86)	-7.52** (-13.8 - -1.22)	-7.53** (-13.9 - -1.21)	19.9*** (14.1 - 25.7)	-0.79 (-7.66 - 6.08)	-0.34 (-7.04 - 6.36)	-0.24 (-6.94 - 6.45)
Log_2 GDP		5.13*** (3.87 - 6.39)	4.80*** (3.43 - 6.16)	4.75*** (3.40 - 6.09)		0.32 (-2.24 - 2.88)	1.23 (-1.53 - 3.99)	1.08 (-1.56 - 3.72)		6.39*** (4.47 - 8.30)	5.52*** (3.47 - 7.57)	5.48*** (3.43 - 7.54)		4.34*** (2.05 - 6.62)	3.29*** (0.85 - 5.73)	3.28*** (0.83 - 5.73)		5.43*** (3.46 - 7.40)	6.27*** (4.16 - 8.38)	6.27*** (4.15 - 8.39)		8.91*** (6.78 - 11.0)	7.62*** (5.38 - 9.87)	7.58*** (5.34 - 9.82)
Older People			0.15 (-0.087 - 0.38)	0.11 (-0.12 - 0.34)			-0.41* (-0.88 - 0.067)	-0.53** (-0.98 - -0.068)			0.39** (0.035 - 0.74)	0.36** (0.0061 - 0.72)			0.47** (0.048 - 0.89)	0.46** (0.036 - 0.88)			-0.37** (-0.73 - -0.010)	-0.37** (-0.73 - -0.00076)			0.57*** (0.19 - 0.96)	0.54*** (0.15 - 0.93)
Women in Parliament				0.094** (0.012 - 0.18)				0.30*** (0.14 - 0.46)				0.065 (-0.060 - 0.19)				0.016 (-0.13 - 0.17)				-0.012 (-0.14 - 0.12)				0.078 (-0.058 - 0.21)
Observations	143	143	143	143	143	143	143	143	143	143	143	143	143	143	143	143	143	143	143	143	143	143	143	143
Adjusted R-squared	0.647	0.757	0.758	0.765	0.327	0.322	0.331	0.389	0.513	0.626	0.636	0.636	0.430	0.478	0.492	0.489	0.284	0.406	0.419	0.415	0.555	0.700	0.715	0.716

Adjusted by Gender:

	Overall				Productivity and Engagement				Well-being				Equity				Cohesion				Security			
VARIABLES	M1	M2	M3	M4	M1	M2	M3	M4	M1	M2	M3	M4	M1	M2	M3	M4	M1	M2	M3	M4	M1	M2	M3	M4
Male	3.85*** (1.23 - 6.47)	3.85*** (2.60 - 5.11)	4.35*** (3.06 - 5.64)	4.27*** (3.00 - 5.54)	9.58*** (6.50 - 12.7)	9.58*** (7.05 - 12.1)	9.10*** (6.46 - 11.7)	8.86*** (6.33 - 11.4)	0.28 (-2.72 - 3.28)	0.28 (-1.53 - 2.08)	1.23 (-0.62 - 3.07)	1.17 (-0.67 - 3.01)	1.69 (-1.58 - 4.97)	1.69 (-0.65 - 4.03)	2.84** (0.45 - 5.24)	2.83** (0.43 - 5.23)	5.43*** (2.71 - 8.15)	5.43*** (3.51 - 7.34)	4.88*** (2.89 - 6.87)	4.87*** (2.88 - 6.86)	2.99 (-1.03 - 7.00)	2.99*** (0.79 - 5.19)	4.23*** (1.99 - 6.46)	4.15*** (1.92 - 6.38)
High Income		1.62 (-2.66 - 5.91)	0.82 (-3.46 - 5.10)	0.91 (-3.29 - 5.12)		11.9*** (3.22 - 20.5)	12.7*** (3.94 - 21.4)	12.9*** (4.55 - 21.3)		-0.17 (-6.34 - 5.99)	-1.71 (-7.80 - 4.39)	-1.65 (-7.73 - 4.44)		1.86 (-6.13 - 9.85)	-0.0049 (-7.92 - 7.91)	0.0074 (-7.93 - 7.94)		-6.33* (-12.9 - 0.21)	-5.44 (-12.0 - 1.13)	-5.44 (-12.0 - 1.15)		-0.056 (-7.58 - 7.47)	-2.06 (-9.47 - 5.35)	-1.97 (-9.36 - 5.41)
Mid Income		-4.51*** (-7.22 - -1.80)	-4.35*** (-7.03 - -1.66)	-4.24*** (-6.88 - -1.60)		-3.25 (-8.72 - 2.22)	-3.41 (-8.88 - 2.06)	-3.08 (-8.34 - 2.19)		-4.72** (-8.62 - -0.81)	-4.40** (-8.23 - -0.58)	-4.33** (-8.15 - -0.51)		-7.62*** (-12.7 - -2.56)	-7.24*** (-12.2 - -2.27)	-7.22*** (-12.2 - -2.24)		-7.05*** (-11.2 - -2.91)	-7.23*** (-11.4 - -3.11)	-7.22*** (-11.4 - -3.09)		-0.65 (-5.41 - 4.11)	-0.24 (-4.89 - 4.41)	-0.13 (-4.77 - 4.50)
Log_2 GDP		5.09*** (4.25 - 5.93)	4.63*** (3.73 - 5.52)	4.58*** (3.70 - 5.46)		0.68 (-1.02 - 2.37)	1.13 (-0.70 - 2.95)	0.98 (-0.78 - 2.73)		5.79*** (4.58 - 7.00)	4.91*** (3.63 - 6.18)	4.88*** (3.60 - 6.15)		4.46*** (2.89 - 6.02)	3.39*** (1.73 - 5.05)	3.38*** (1.72 - 5.04)		5.74*** (4.46 - 7.02)	6.25*** (4.87 - 7.62)	6.24*** (4.86 - 7.62)		8.71*** (7.24 - 10.2)	7.56*** (6.01 - 9.12)	7.52*** (5.97 - 9.06)
Older People			0.21*** (0.056 - 0.35)	0.17** (0.024 - 0.32)			-0.20 (-0.51 - 0.10)	-0.30** (-0.59 - -0.0014)			0.39*** (0.18 - 0.60)	0.37*** (0.16 - 0.58)			0.48*** (0.20 - 0.75)	0.47*** (0.19 - 0.75)			-0.23* (-0.46 - 0.0033)	-0.23* (-0.46 - 0.0035)			0.51*** (0.25 - 0.77)	0.48*** (0.22 - 0.74)
Women in Parliament				0.088*** (0.034 - 0.14)				0.26*** (0.16 - 0.37)				0.058 (-0.020 - 0.14)				0.012 (-0.090 - 0.11)				0.0062 (-0.078 - 0.091)				0.085* (-0.0096 - 0.18)
Observations	286	286	286	286	286	286	286	286	286	286	286	286	286	286	286	286	286	286	286	286	286	286	286	286
Adjusted R-squared	0.025	0.777	0.781	0.789	0.113	0.403	0.404	0.448	-0.003	0.637	0.652	0.653	0.000	0.490	0.508	0.506	0.048	0.527	0.532	0.530	0.004	0.700	0.715	0.717

Note: *** p<0.01, ** p<0.05, * p<0.1.
- **High-income** and **middle-income** dummy variables was compared to low-income. **GDP** per capita was adjusted for purchasing power parity. **Older people** refer to the proportion of 65+ in the population. **Women in Parliament** refers to the proportion of seats women hold in the national parliament.
- For regression models that were adjusted by gender, scores from each country by gender were used.

Supplementary figure S1A. Scatterplot and correlation coefficient (Spearman's correlation test) between domain scores for **males**

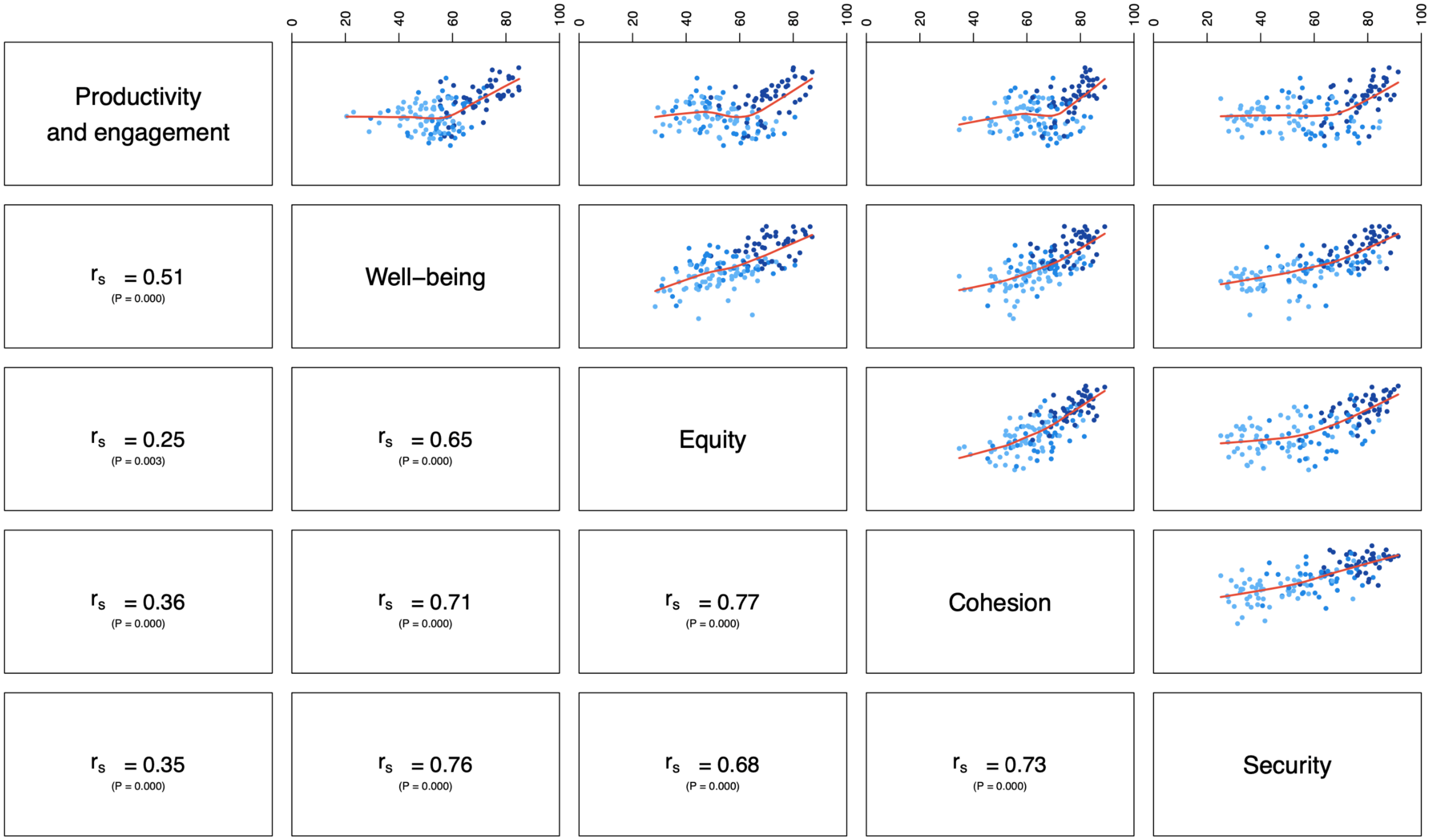

Note: P-value is one sided.

Supplementary figure S1B. Scatterplot and correlation coefficient (Spearman's correlation test) between domain scores for **females**

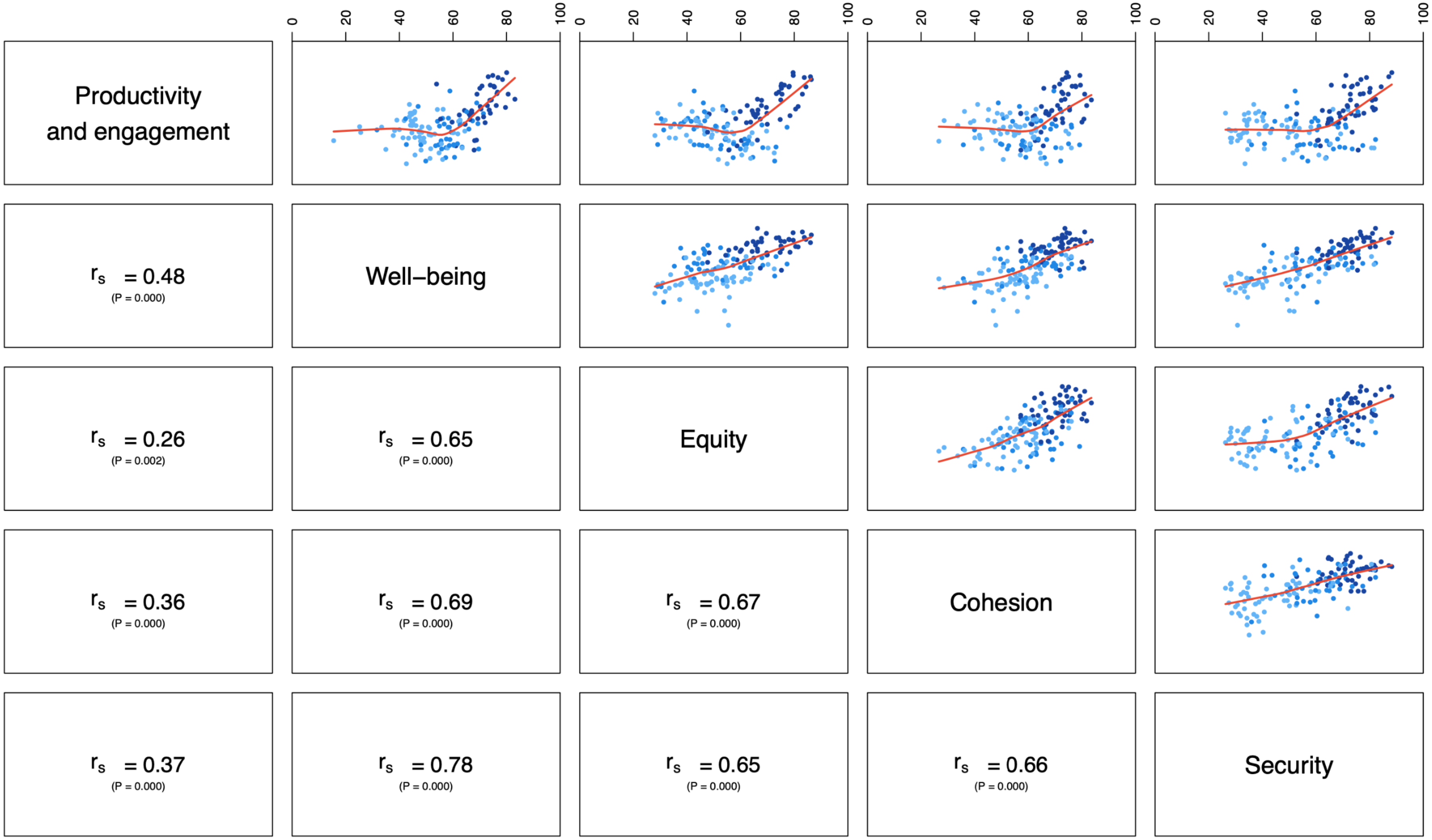

Note: P-value is one sided.

Supplementary figure S2. Measures in well-being domain

A. Healthy life expectancy

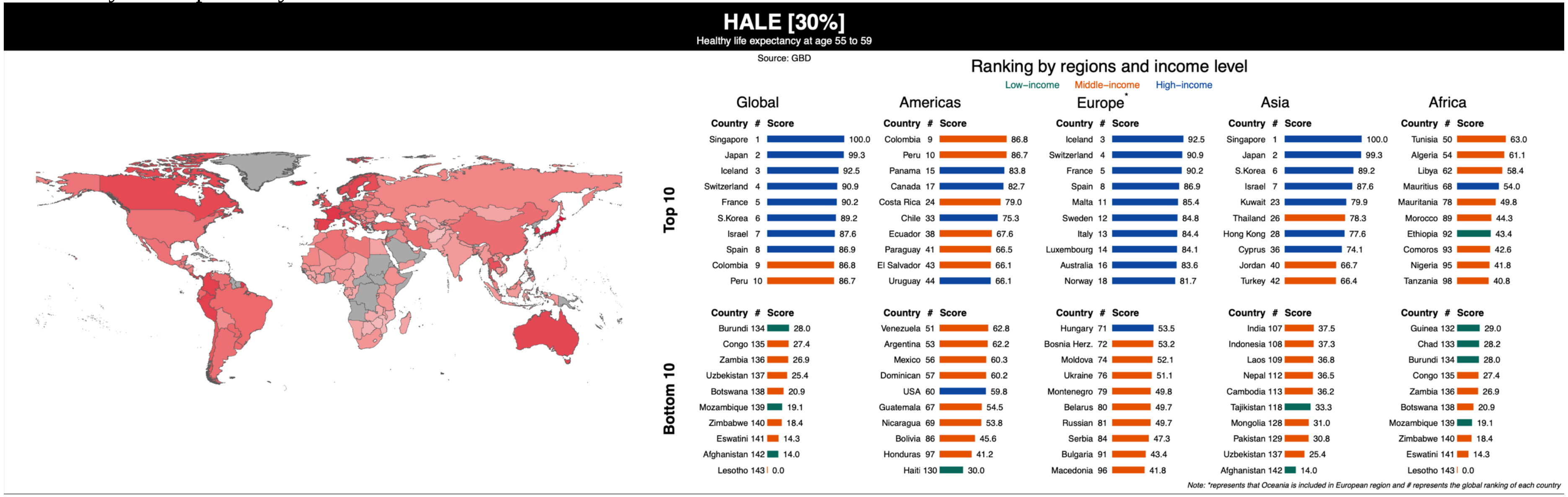

B. Share of healthy life expectancy to overall life expectancy

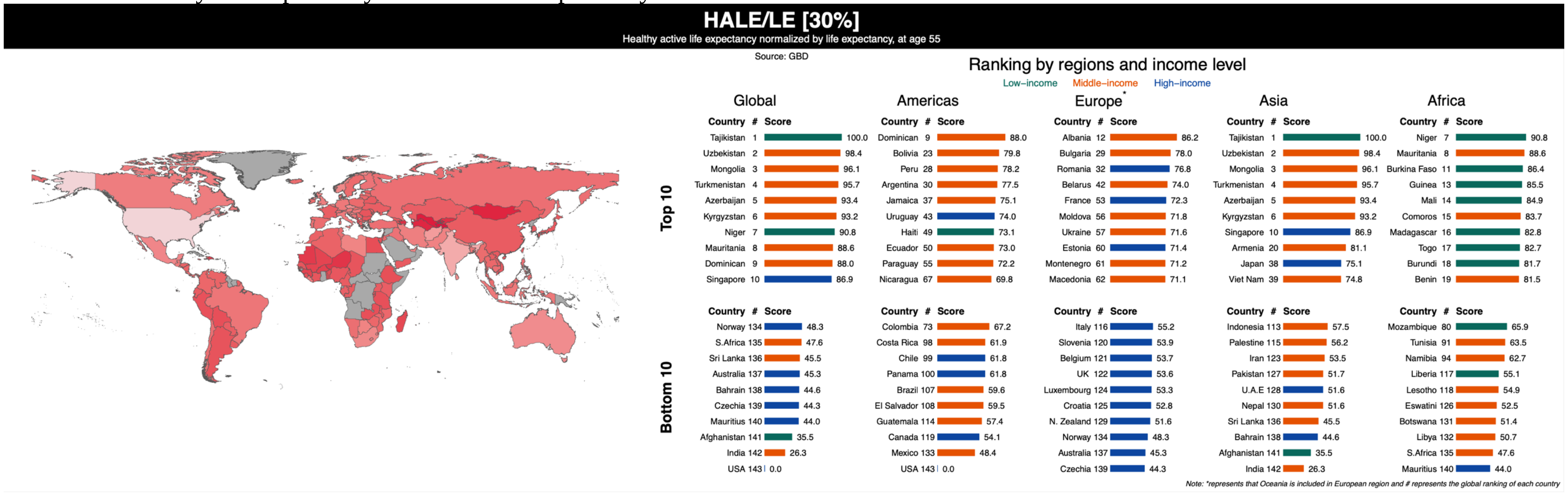

C. Life satisfaction

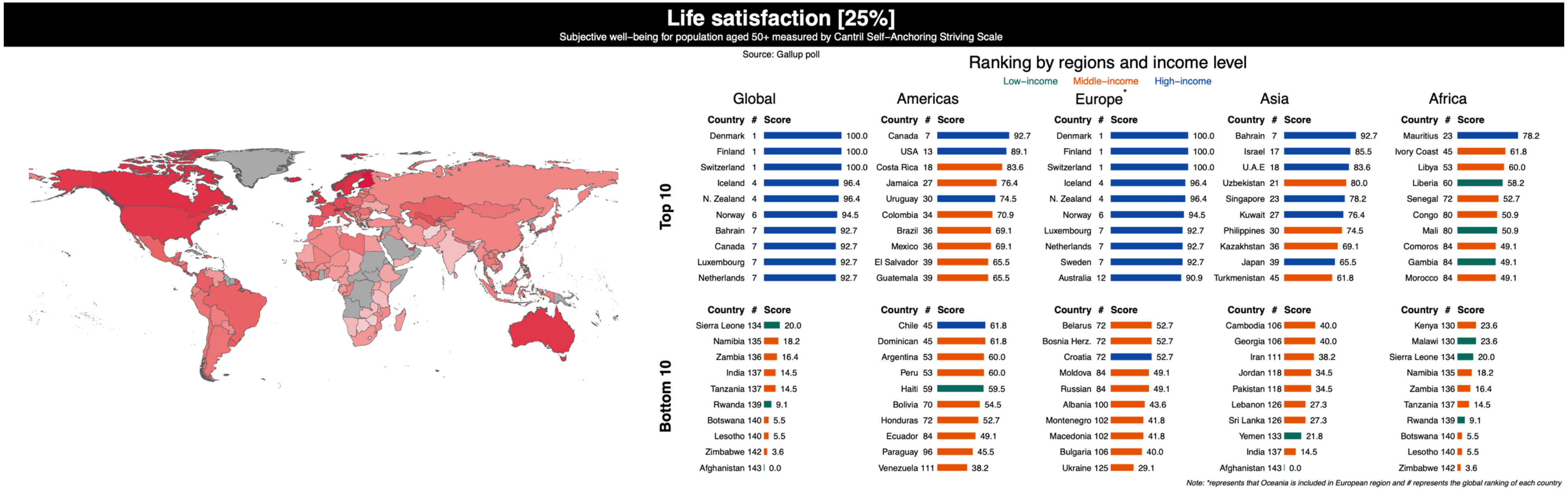

D. Universal Health Coverage

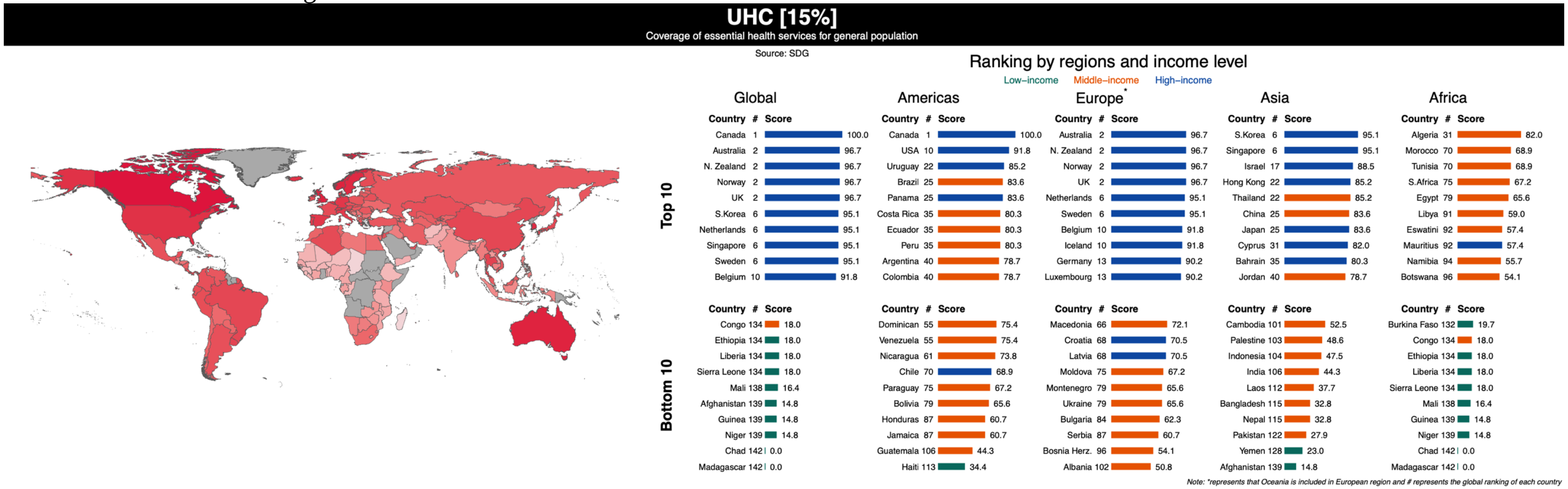

E. Well-being domain

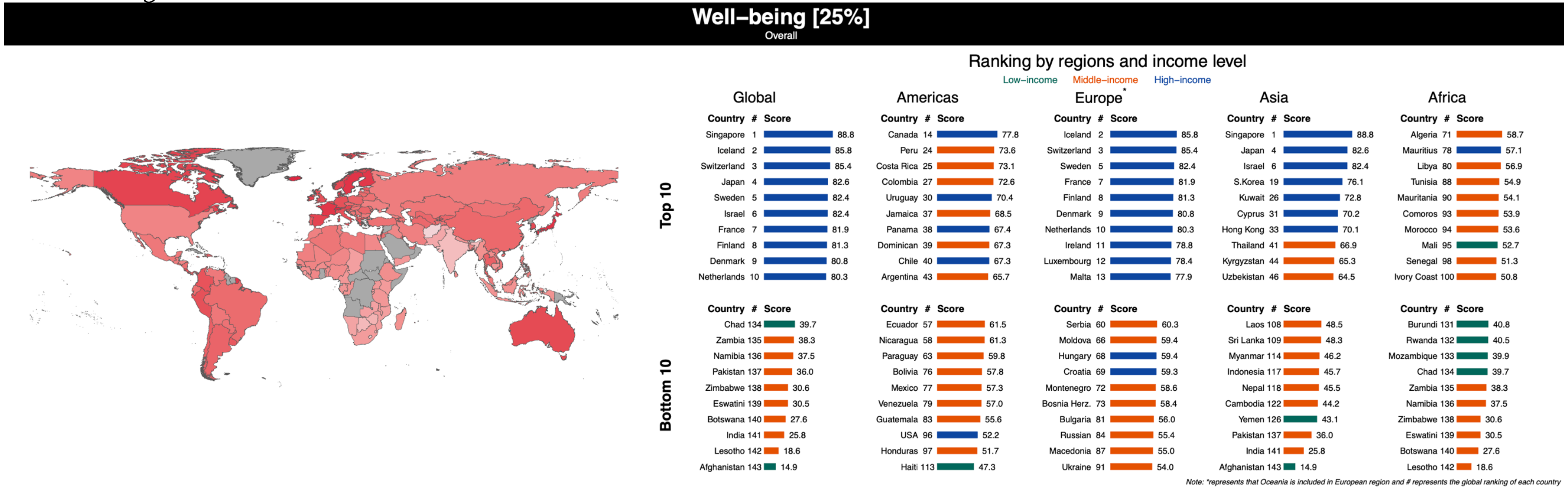

Supplementary figure S3. Measures in productivity and engagement domain

A. Labour Force Participation Rate

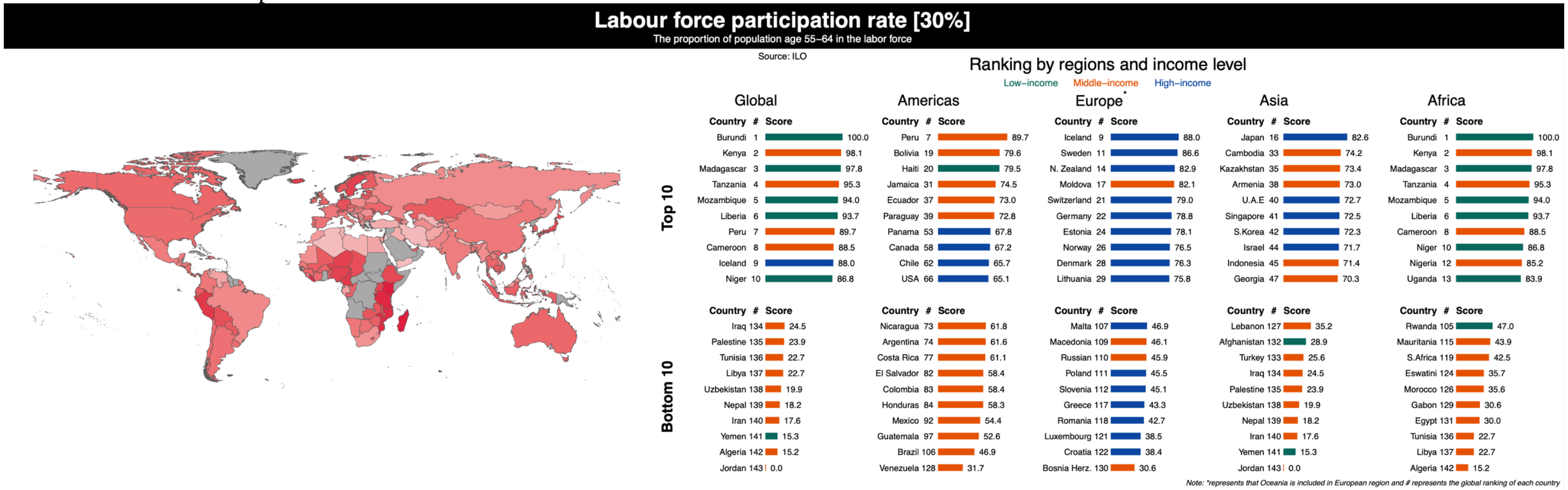

B. Retraining

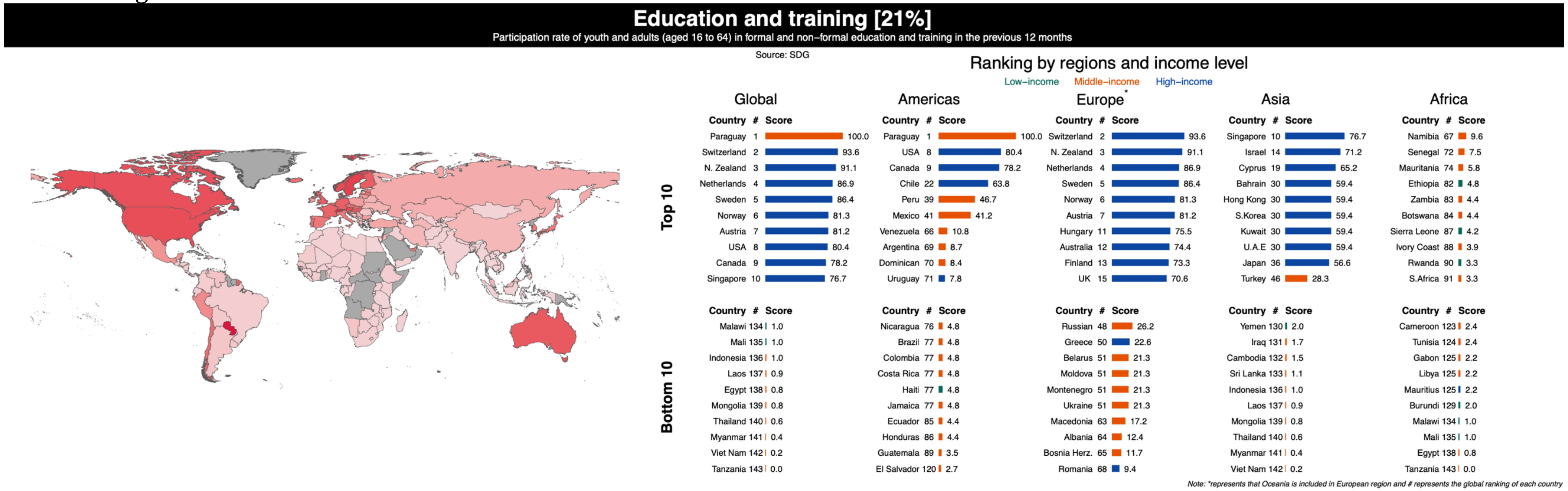

C. Volunteering

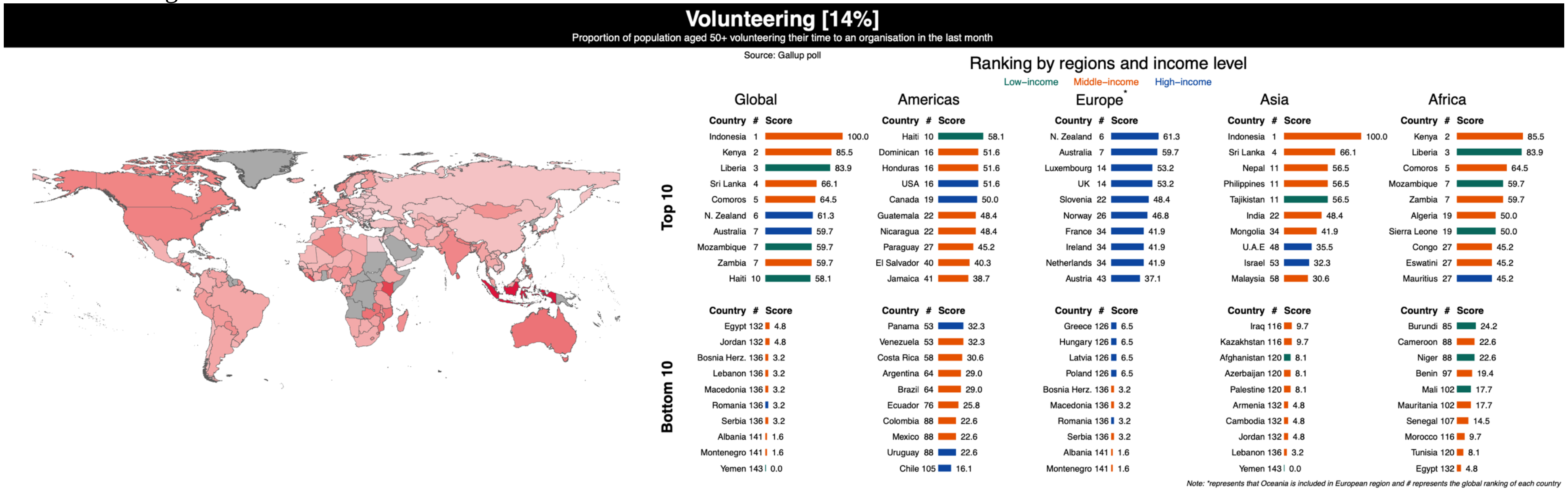

D. Active and Productive

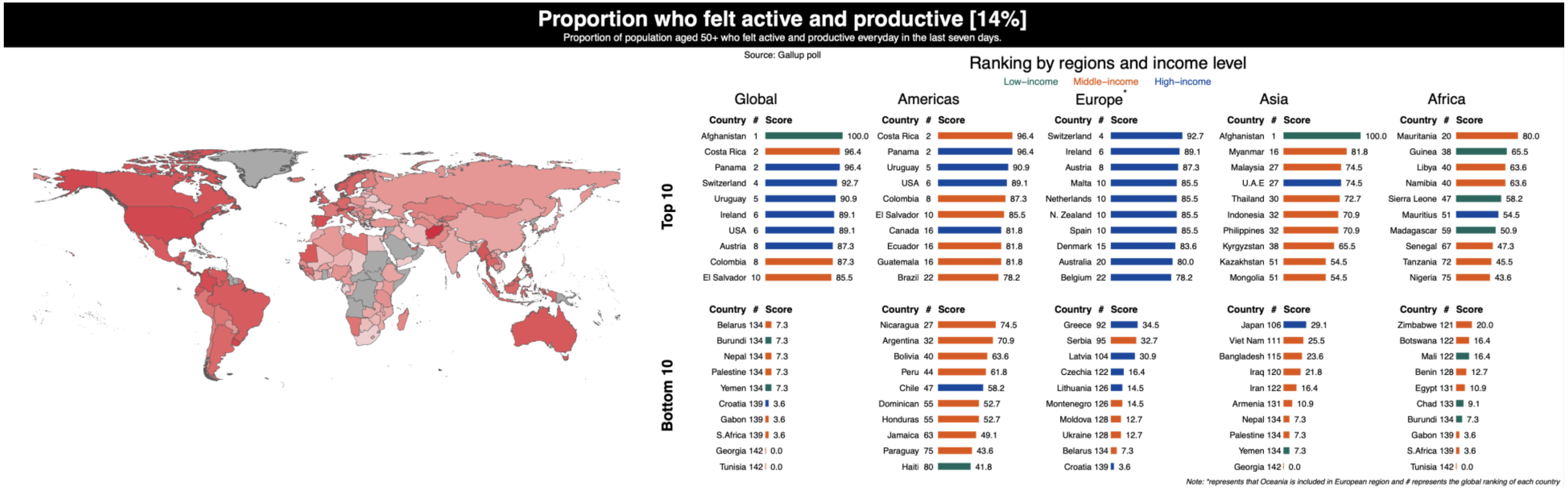

E. Having an ideal job

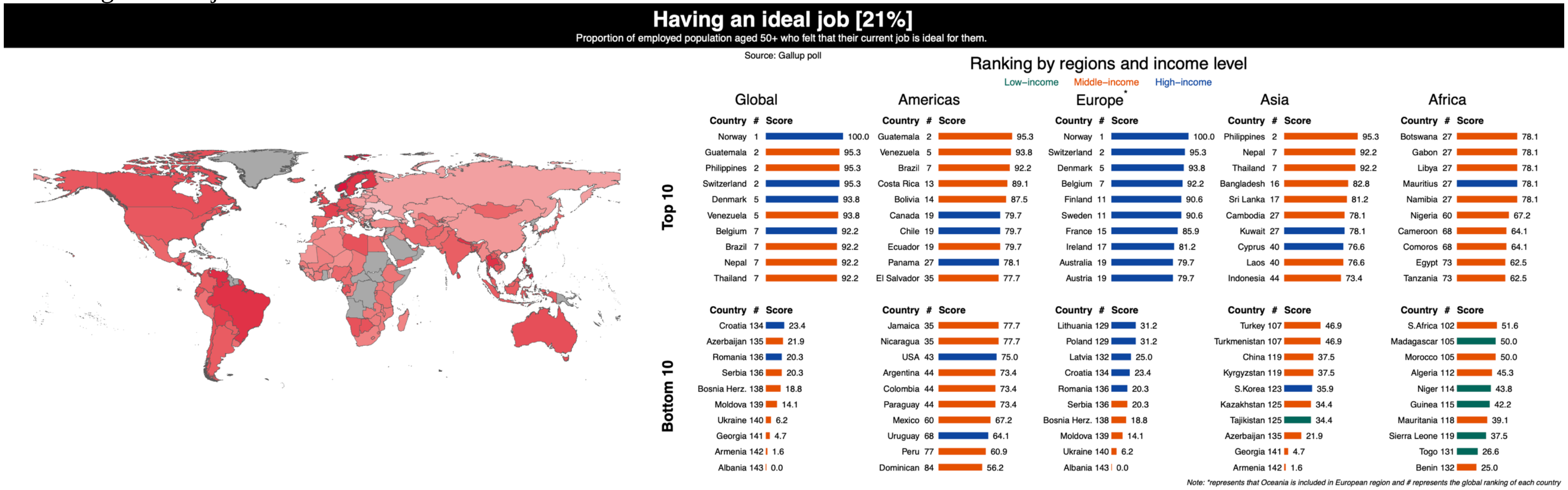

F. Productivity and engagement domain

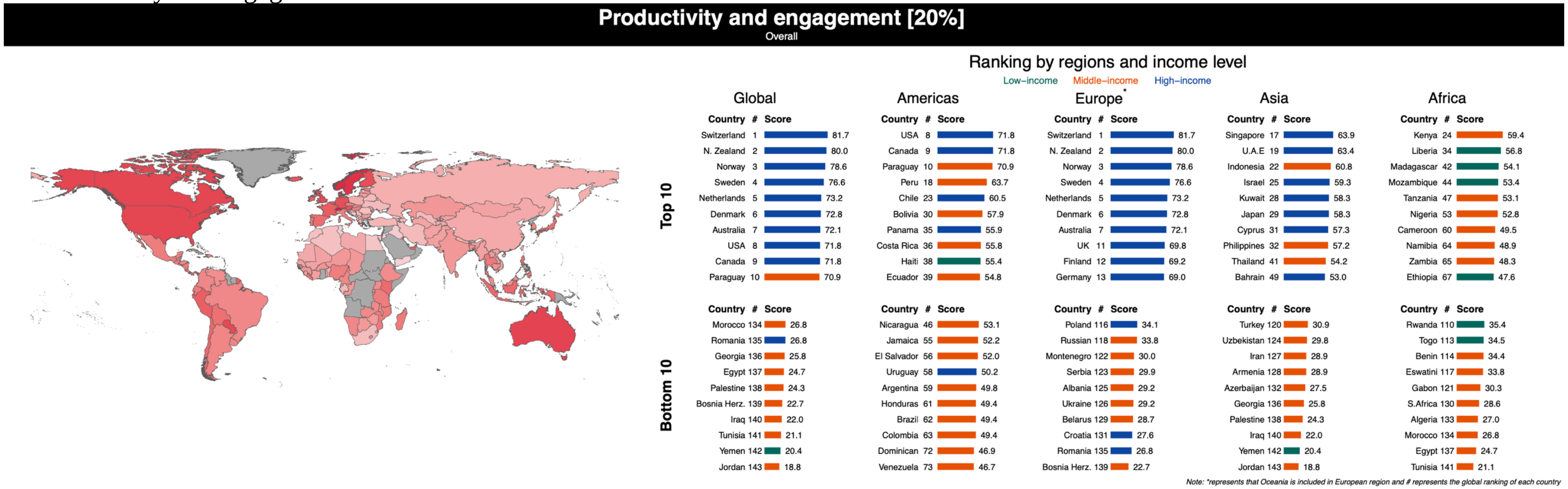

Supplementary figure S4. Measures in equity domain

A. 1-Gini coefficient

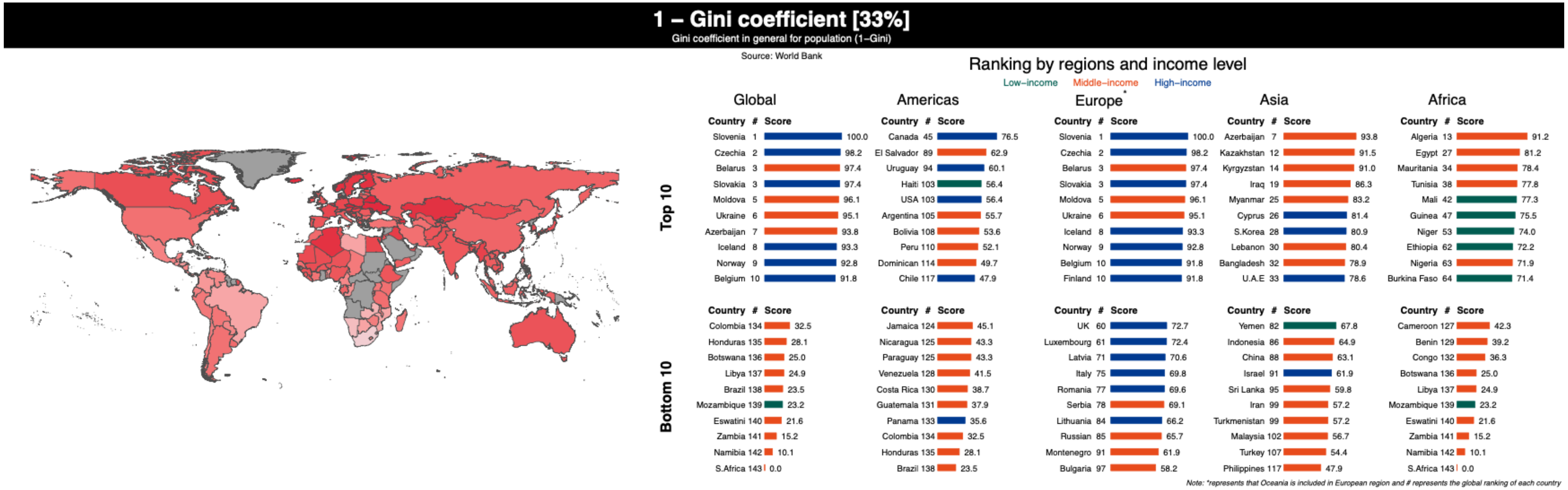

B. Living comfortably or getting by on current income, age 50+

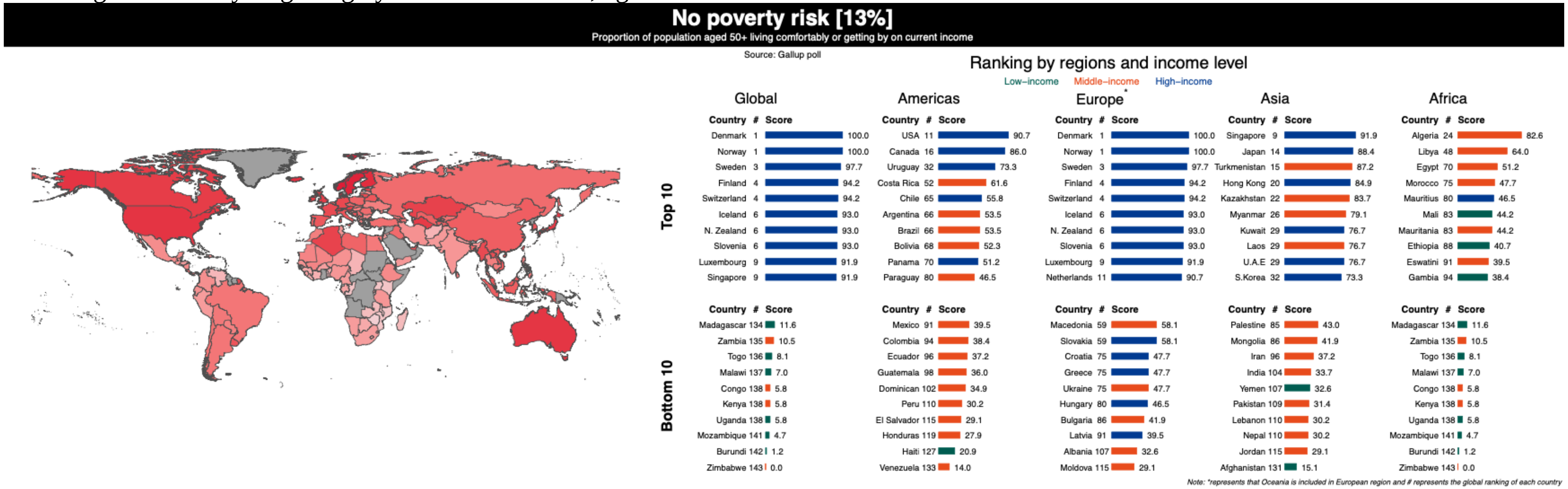

C. Living comfortably or getting by on current income, age 50+ relative to age 15-49

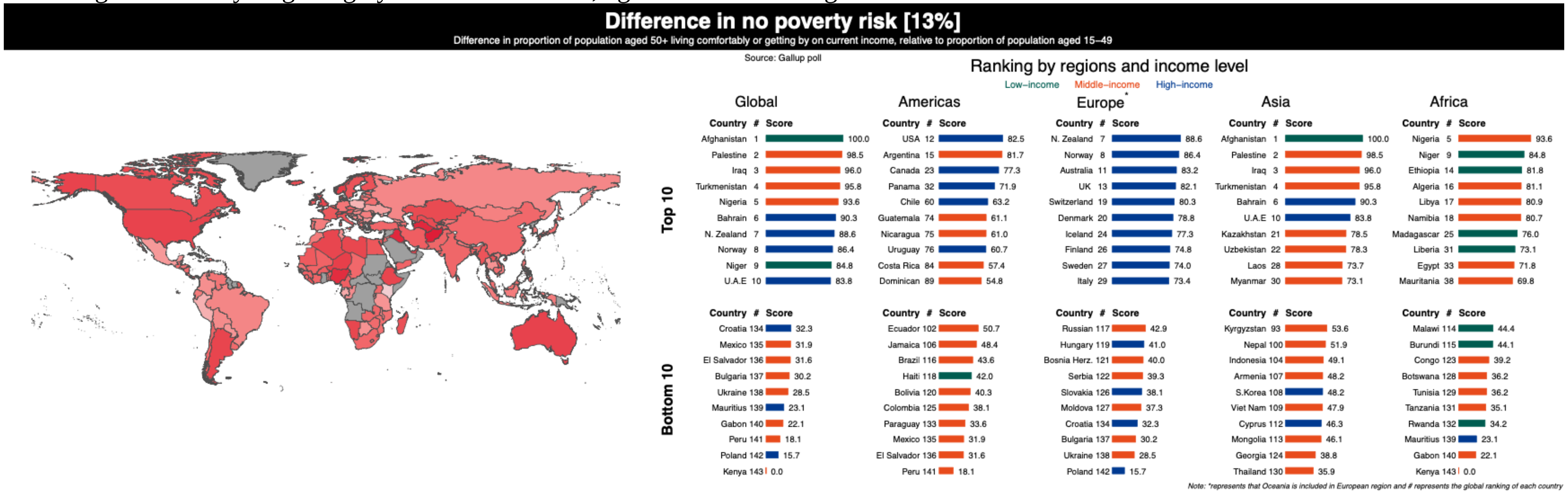

D. Food security, age 50+

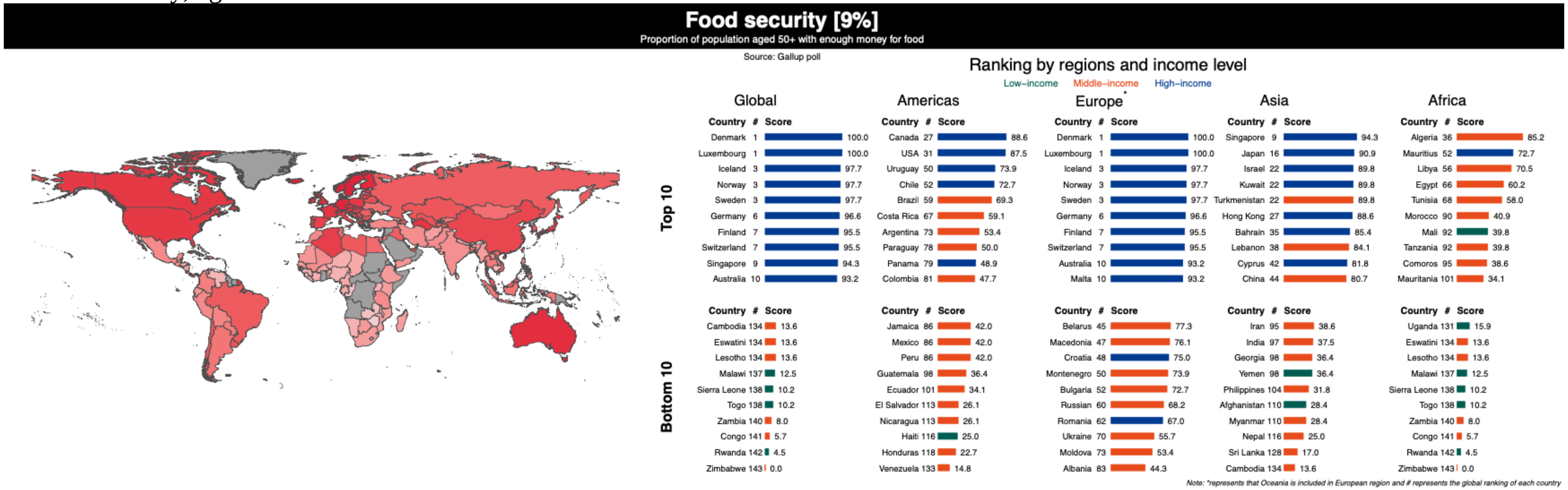

E. Food security, age 50+ relative to age 15-49

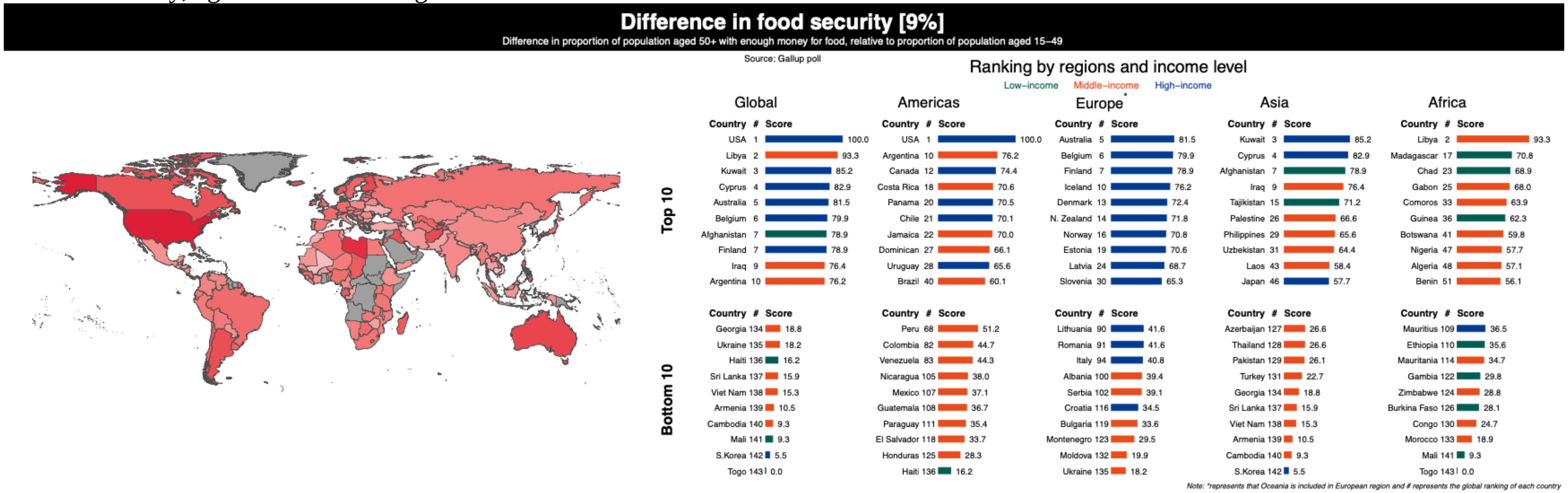

F. High school attainment, age 55 to 64

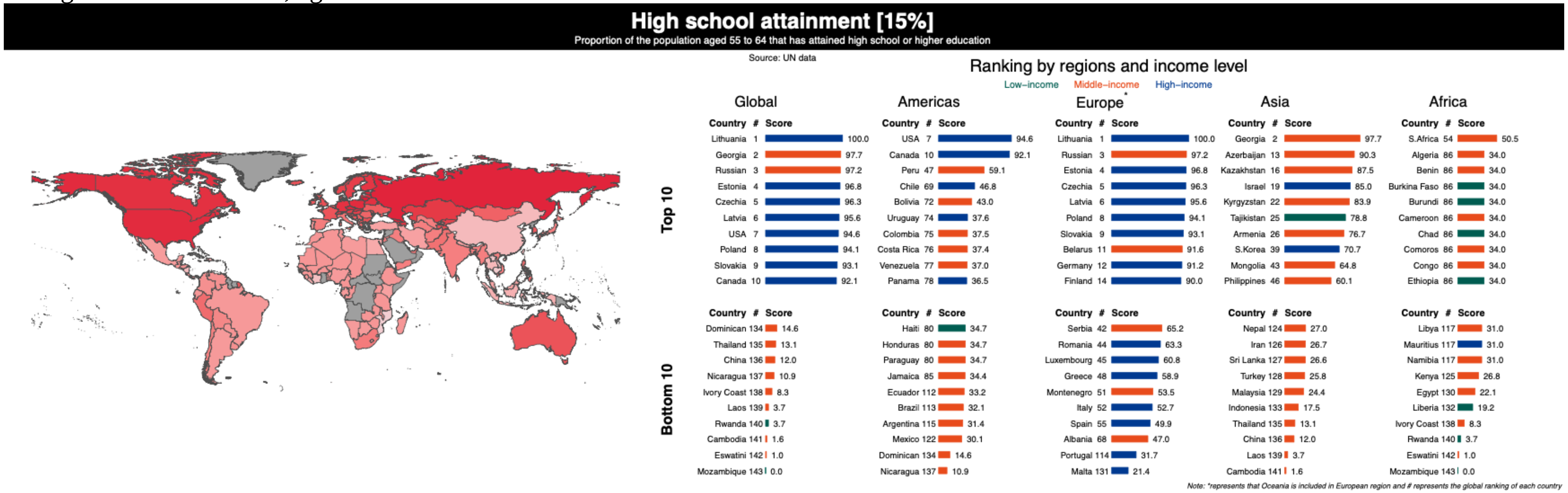

G. Difference in LFPR

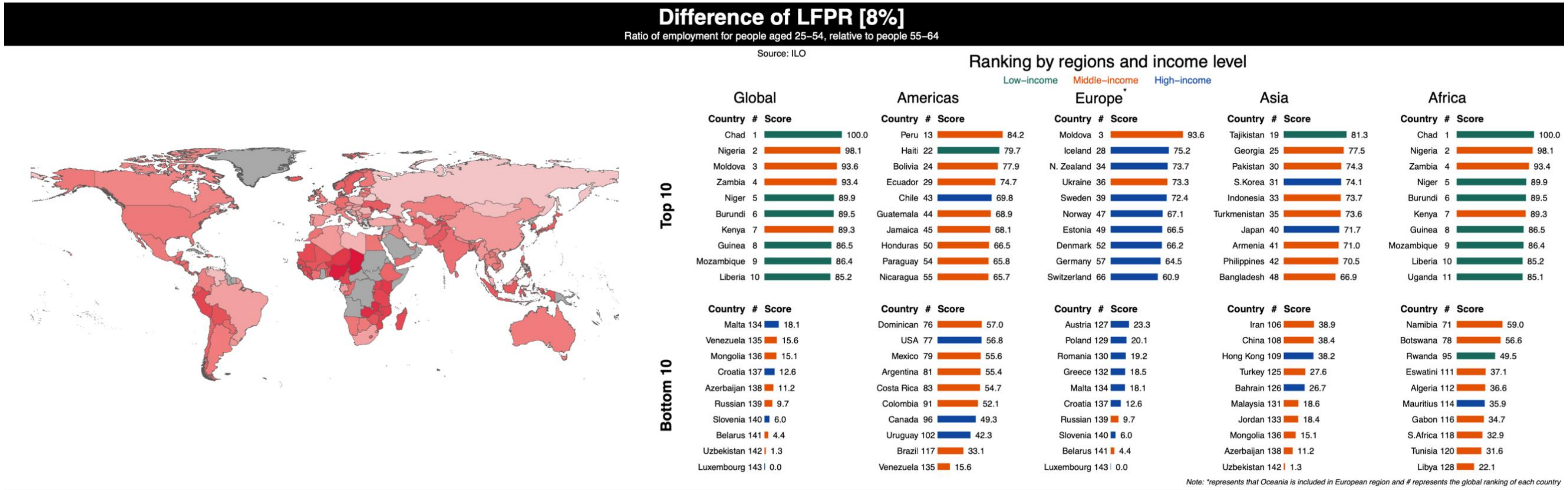

H. Equity domain

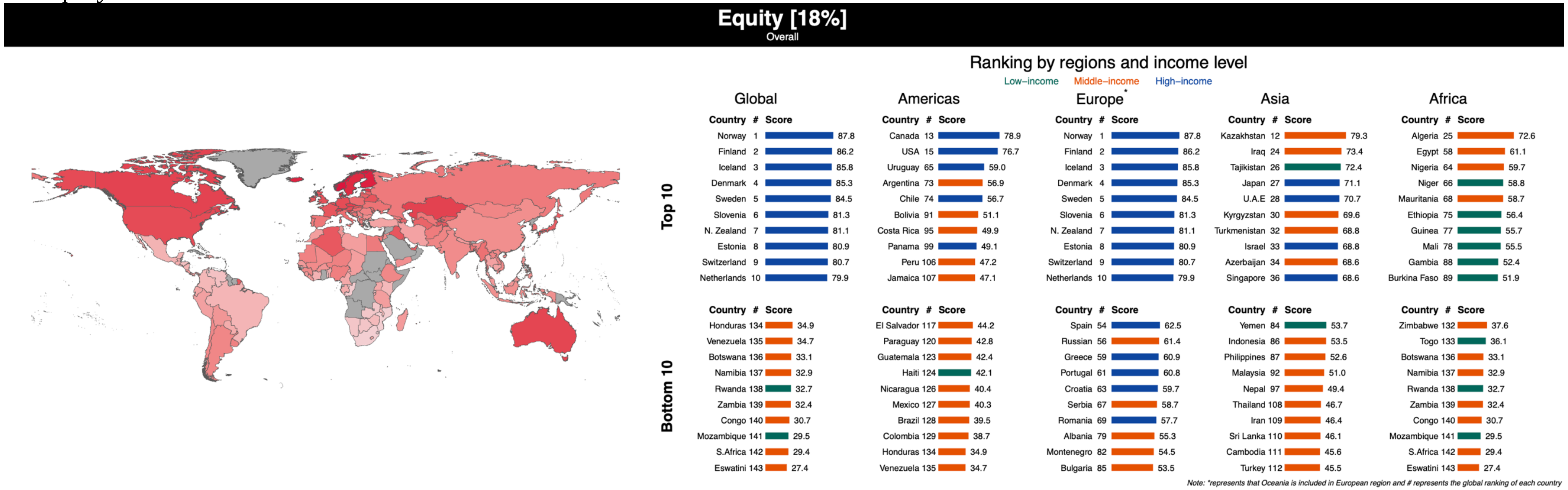

Supplementary figure S5. Measures in cohesion domain

A. Trust neighbour

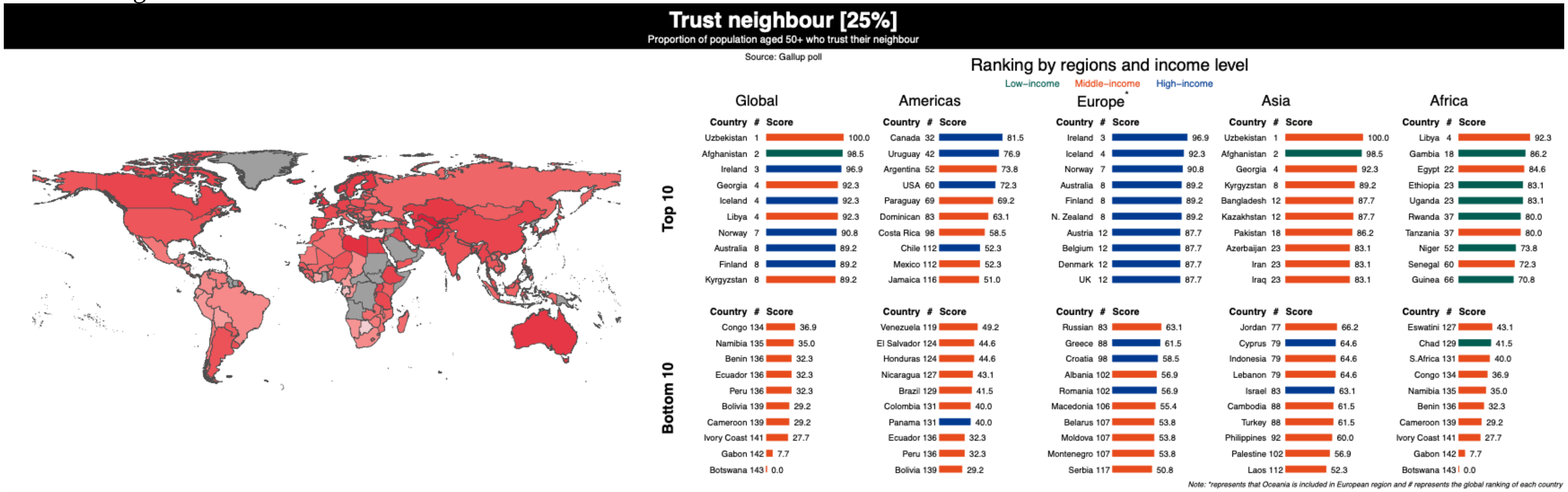

B. Social support

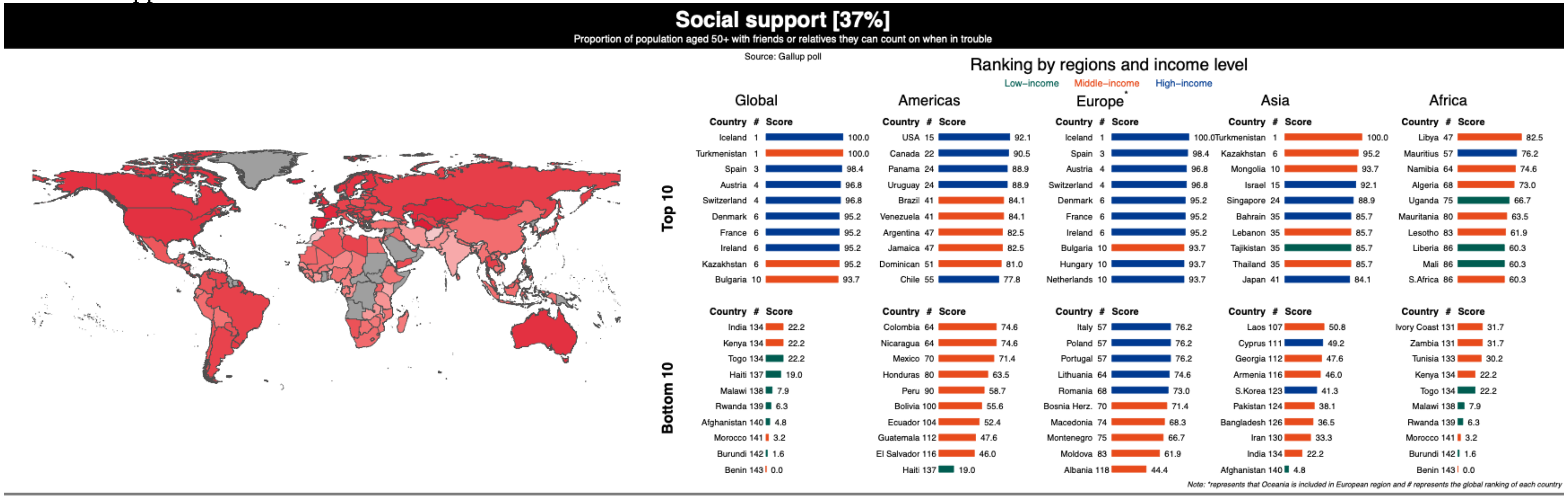

C. Co-residence

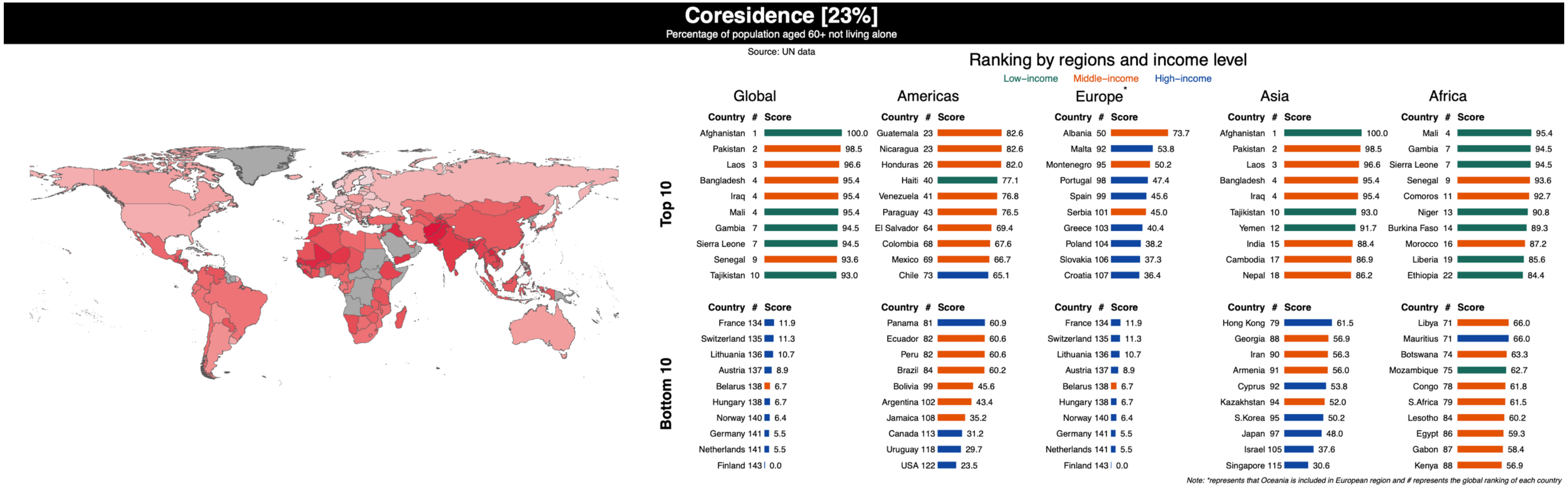

D. Technology

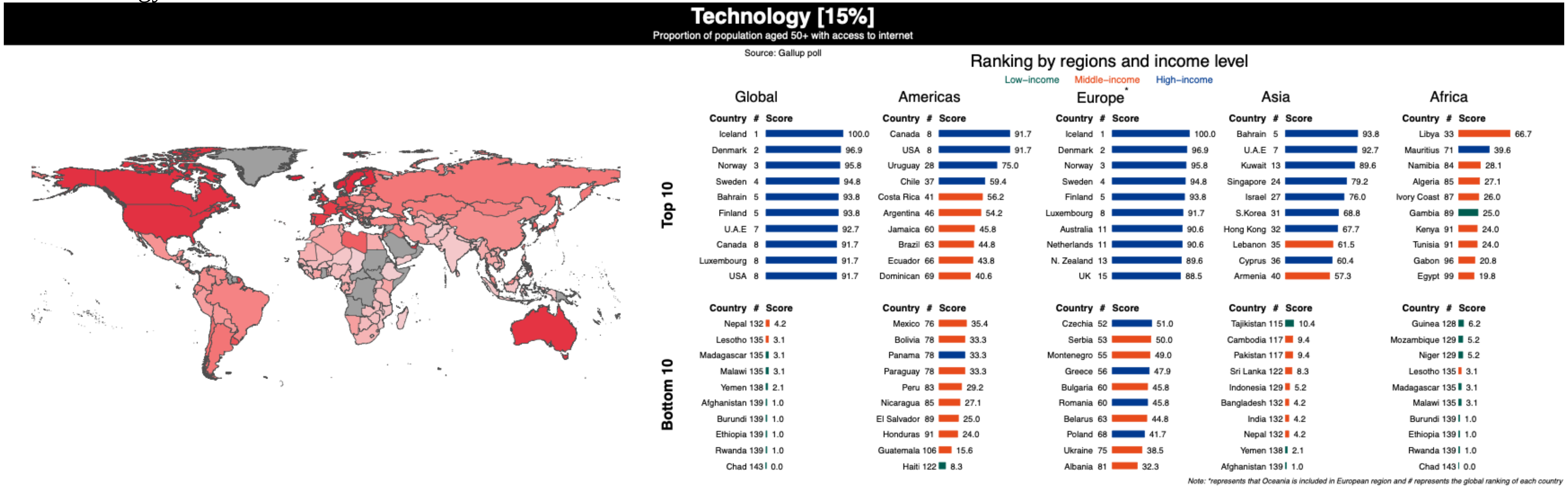

E. Cohesion domain

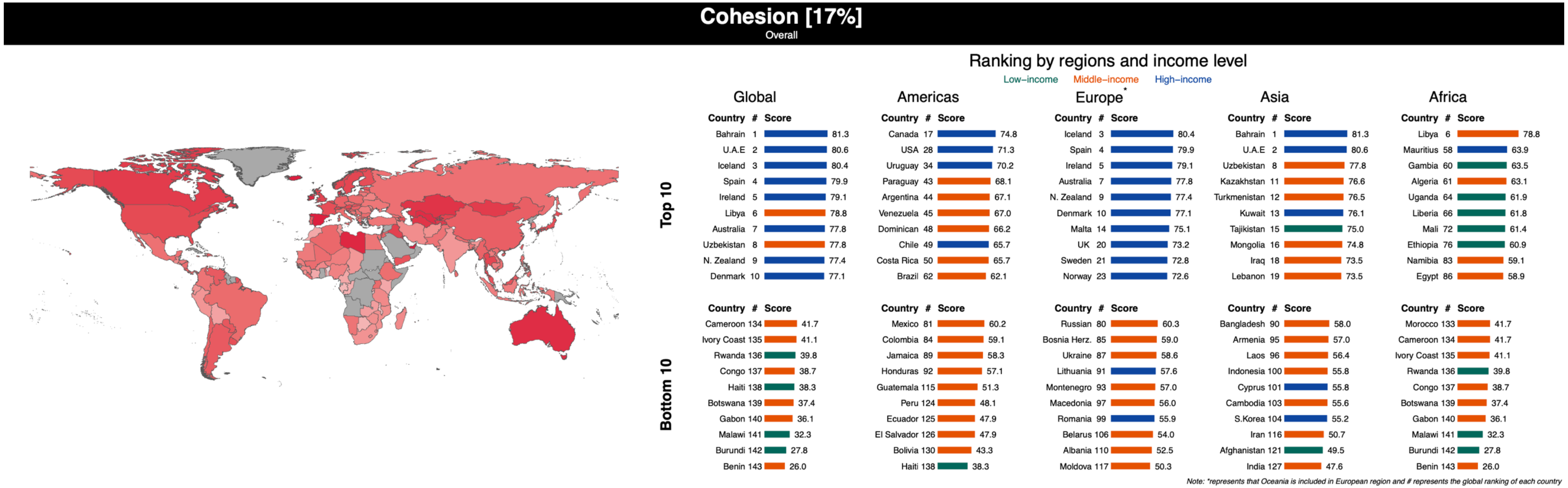

Supplementary figure S6. Measures in security domain

A. Income

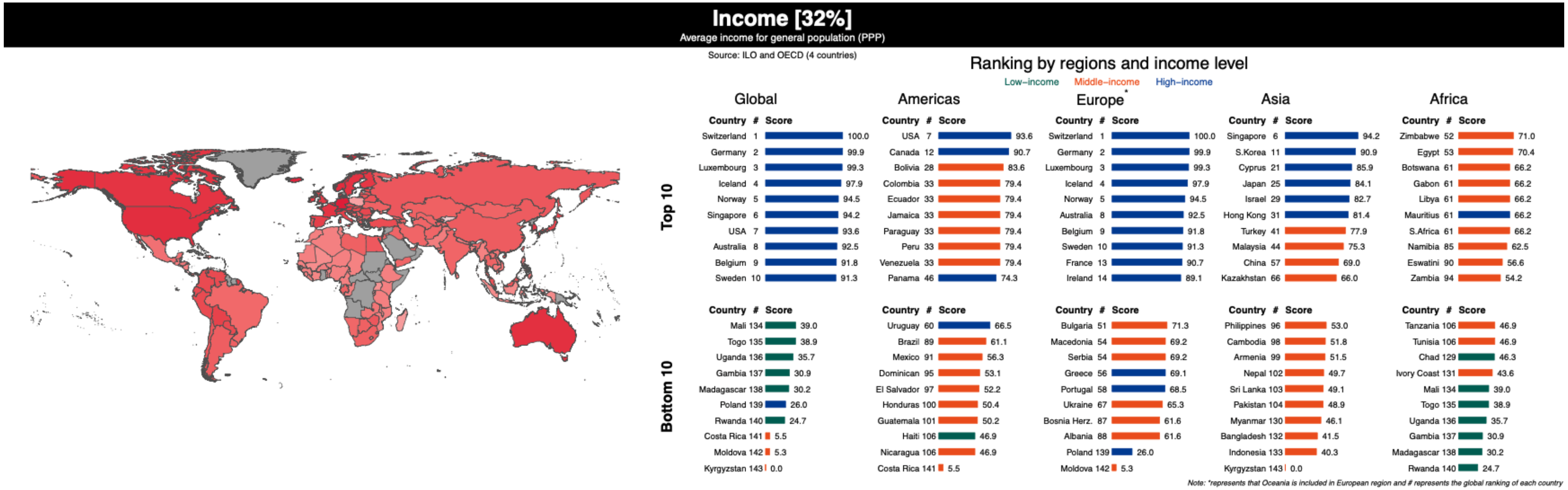

B. Pension

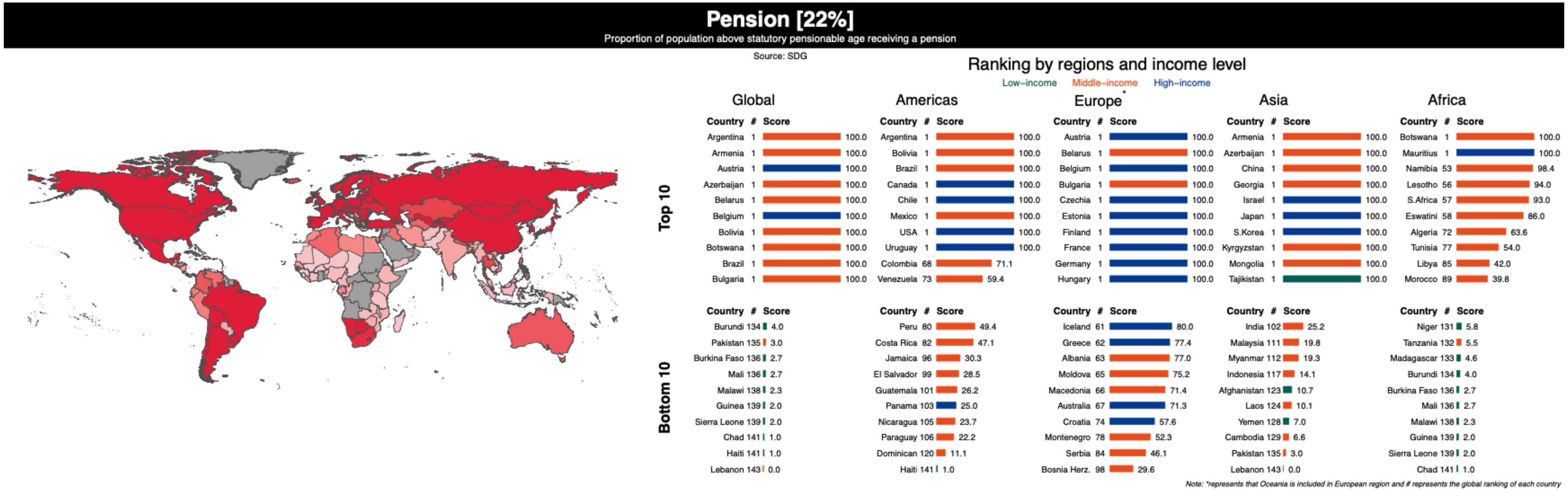

C. Physical safety

Physical safety [9%]

Proportion of population aged 50+ who feel safe walking alone at night in the city they live in

Source: Gallup poll

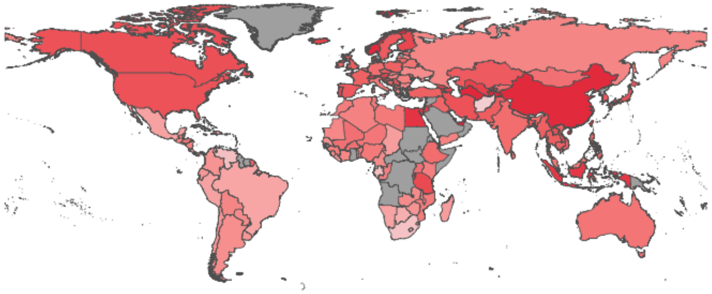

Ranking by regions and income level

Low–income Middle–income High–income

Top 10

Global Country	#	Score	Americas Country	#	Score	Europe* Country	#	Score	Asia Country	#	Score	Africa Country	#	Score
Singapore	1	100.0	Canada	29	75.6	Norway	6	94.9	Singapore	1	100.0	Egypt	8	91.0
Tajikistan	2	97.4	USA	30	74.4	Slovenia	9	88.5	Tajikistan	2	97.4	Rwanda	25	78.2
U.A.E	2	97.4	Jamaica	70	57.7	Switzerland	9	88.5	U.A.E	2	97.4	Tanzania	25	78.2
Kuwait	4	96.2	Haiti	80	53.8	Denmark	13	84.6	Kuwait	4	96.2	Burundi	27	76.9
Turkmenistan	4	96.2	Honduras	82	52.6	Austria	18	83.3	Turkmenistan	4	96.2	Comoros	52	65.4
China	6	94.9	El Salvador	85	51.3	Iceland	18	83.3	China	6	94.9	Mauritius	62	61.5
Norway	6	94.9	Guatemala	98	46.2	Finland	21	80.8	Armenia	9	88.5	Ethiopia	67	59.0
Egypt	8	91.0	Nicaragua	104	44.9	Luxembourg	21	80.8	Indonesia	9	88.5	Burkina Faso	74	56.4
Armenia	9	88.5	Bolivia	108	43.6	Portugal	27	76.9	Azerbaijan	13	84.6	Mali	74	56.4
Indonesia	9	88.5	Paraguay	116	39.7	Netherlands	35	73.1	Georgia	13	84.6	Uganda	74	56.4

Bottom 10

Global Country	#	Score	Americas Country	#	Score	Europe* Country	#	Score	Asia Country	#	Score	Africa Country	#	Score
Dominican	134	25.6	Chile	124	34.6	Italy	82	52.6	Philippines	62	61.5	Zimbabwe	122	35.9
Namibia	134	25.6	Ecuador	125	33.3	Hungary	95	47.4	Sri Lanka	62	61.5	Chad	127	29.5
Brazil	136	24.4	Uruguay	125	33.3	Belgium	98	46.2	India	67	59.0	Madagascar	127	29.5
Eswatini	137	23.1	Costa Rica	127	29.5	Romania	98	46.2	Kyrgyzstan	70	57.7	Liberia	131	28.2
Botswana	138	19.2	Panama	127	29.5	Bulgaria	104	44.9	Mongolia	74	56.4	Namibia	134	25.6
Lesotho	139	16.7	Mexico	132	26.9	Latvia	108	43.6	Nepal	74	56.4	Eswatini	137	23.1
Gabon	140	9.0	Peru	132	26.9	Russian	108	43.6	Malaysia	85	51.3	Botswana	138	19.2
Venezuela	140	9.0	Dominican	134	25.6	Slovakia	108	43.6	Lebanon	92	48.7	Lesotho	139	16.7
S.Africa	142	6.4	Brazil	136	24.4	Greece	112	42.3	Yemen	98	46.2	Gabon	140	9.0
Afghanistan	143	0.0	Venezuela	140	9.0	Ukraine	116	39.7	Afghanistan	143	0.0	S.Africa	142	6.4

*Note: *represents that Oceania is included in European region and # represents the global ranking of each country*

D. No harm from mental health issues, age 50+

No harm from mental health issues [20%]

Proportion of population aged 50+ who did not experience harm from mental health issues

Source: Gallup poll

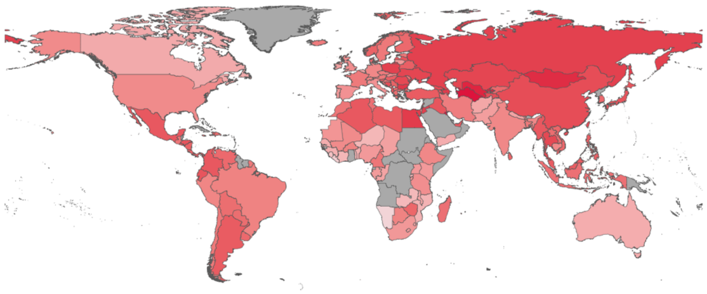

Ranking by regions and income level

Low–income Middle–income High–income

Top 10

Global Country	#	Score	Americas Country	#	Score	Europe* Country	#	Score	Asia Country	#	Score	Africa Country	#	Score
Kuwait	1	100.0	Ecuador	21	76.9	Bulgaria	5	90.4	Kuwait	1	100.0	Egypt	6	88.5
Turkmenistan	1	100.0	Honduras	21	76.9	Poland	9	86.5	Turkmenistan	1	100.0	Algeria	28	75.0
Uzbekistan	3	96.2	Colombia	28	75.0	Russian	9	86.5	Uzbekistan	3	96.2	Libya	35	73.1
Mongolia	4	94.2	Mexico	28	75.0	Ukraine	12	84.6	Mongolia	4	94.2	Zimbabwe	53	67.3
Bulgaria	5	90.4	Argentina	35	73.1	Romania	15	82.7	Azerbaijan	6	88.5	Madagascar	69	61.5
Azerbaijan	6	88.5	El Salvador	35	73.1	Bosnia Herz.	18	80.8	Singapore	6	88.5	Tunisia	69	61.5
Egypt	6	88.5	Panama	40	71.2	Albania	21	76.9	Jordan	9	86.5	Cameroon	72	59.6
Singapore	6	88.5	Haiti	46	70.7	Latvia	21	76.9	Kazakhstan	12	84.6	Morocco	72	59.6
Jordan	9	86.5	Costa Rica	47	69.2	Slovenia	28	75.0	Palestine	12	84.6	Mauritius	81	55.8
Poland	9	86.5	Nicaragua	47	69.2	Belarus	40	71.2	Georgia	15	82.7	Botswana	87	51.9

Bottom 10

Global Country	#	Score	Americas Country	#	Score	Europe* Country	#	Score	Asia Country	#	Score	Africa Country	#	Score
Mozambique	134	21.2	Bolivia	63	63.5	Italy	103	42.3	Indonesia	69	61.5	Mozambique	134	21.2
Togo	134	21.2	Dominican	63	63.5	Spain	103	42.3	Bangladesh	81	55.8	Togo	134	21.2
Ivory Coast	136	19.2	Jamaica	63	63.5	N. Zealand	105	40.4	Iran	86	53.8	Ivory Coast	136	19.2
Niger	136	19.2	Paraguay	63	63.5	Portugal	107	38.5	Cambodia	93	48.1	Niger	136	19.2
Gambia	138	17.3	Guatemala	72	59.6	Lithuania	114	36.5	India	93	48.1	Gambia	138	17.3
Rwanda	138	17.3	Chile	78	57.7	Ireland	118	32.7	Israel	93	48.1	Rwanda	138	17.3
Zambia	138	17.3	Brazil	87	51.9	Luxembourg	118	32.7	Pakistan	116	34.6	Zambia	138	17.3
Sierra Leone	141	11.5	Peru	90	50.0	Belgium	124	28.8	Nepal	121	30.8	Sierra Leone	141	11.5
Liberia	142	3.8	USA	98	46.2	Netherlands	124	28.8	Afghanistan	124	28.8	Liberia	142	3.8
Namibia	143	0.0	Canada	128	26.9	Australia	129	25.0	Yemen	129	25.0	Namibia	143	0.0

*Note: *represents that Oceania is included in European region and # represents the global ranking of each country*

E. Satisfaction with quality healthcare

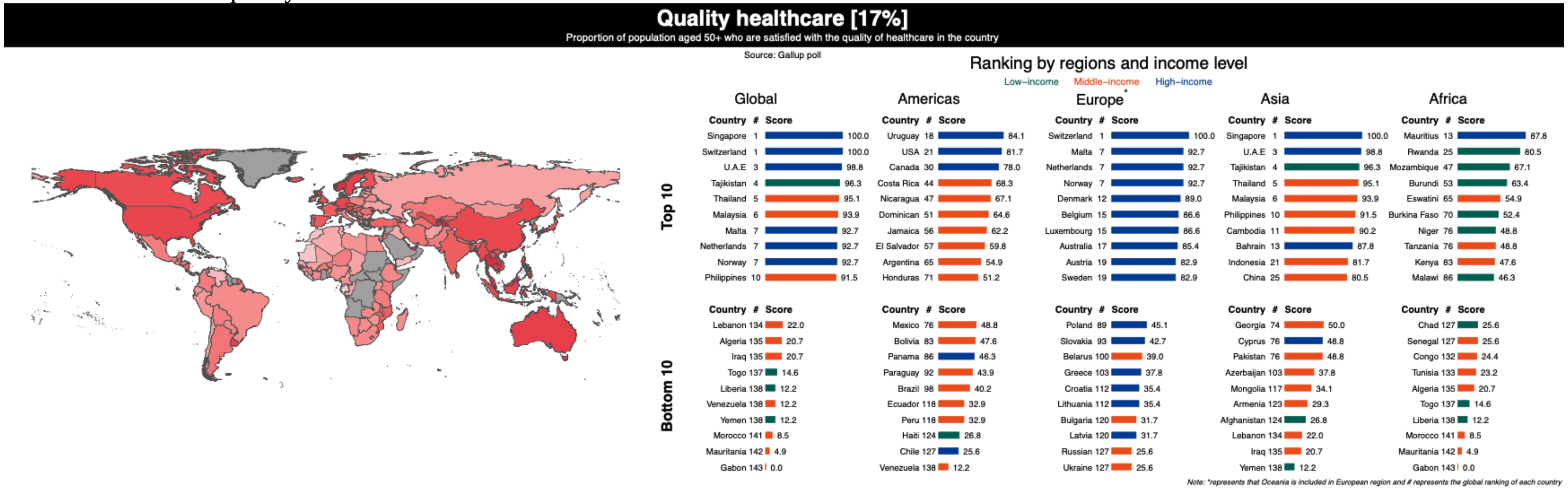

F. Security domain

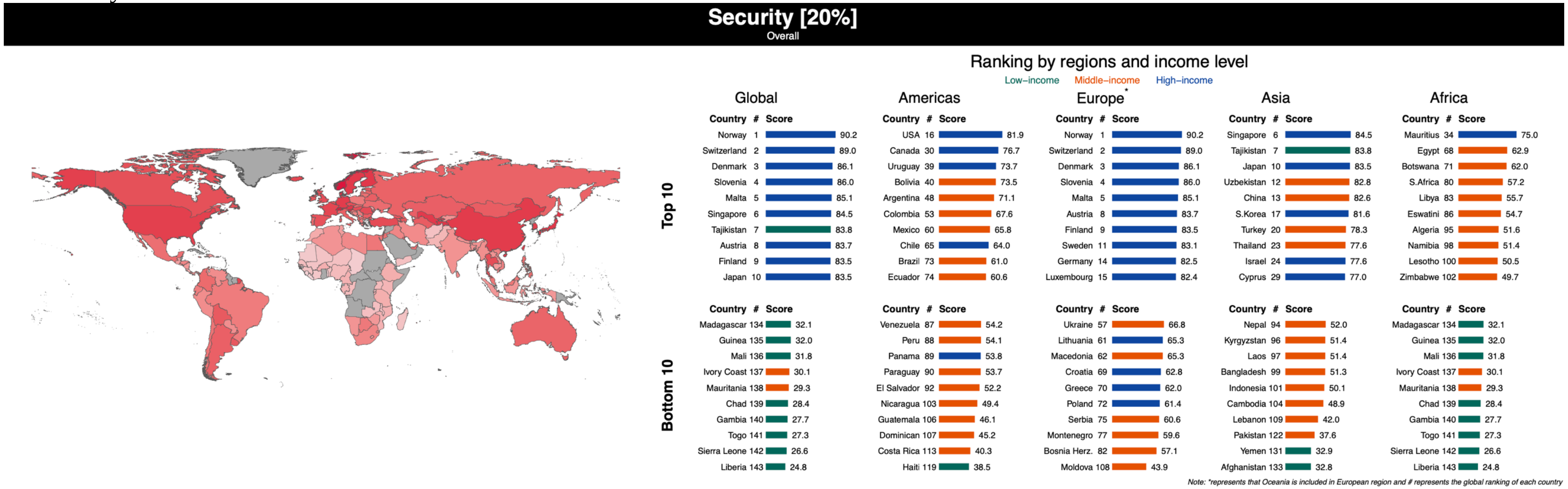

Supplementary figure S7. Scores for Overall Aging Index

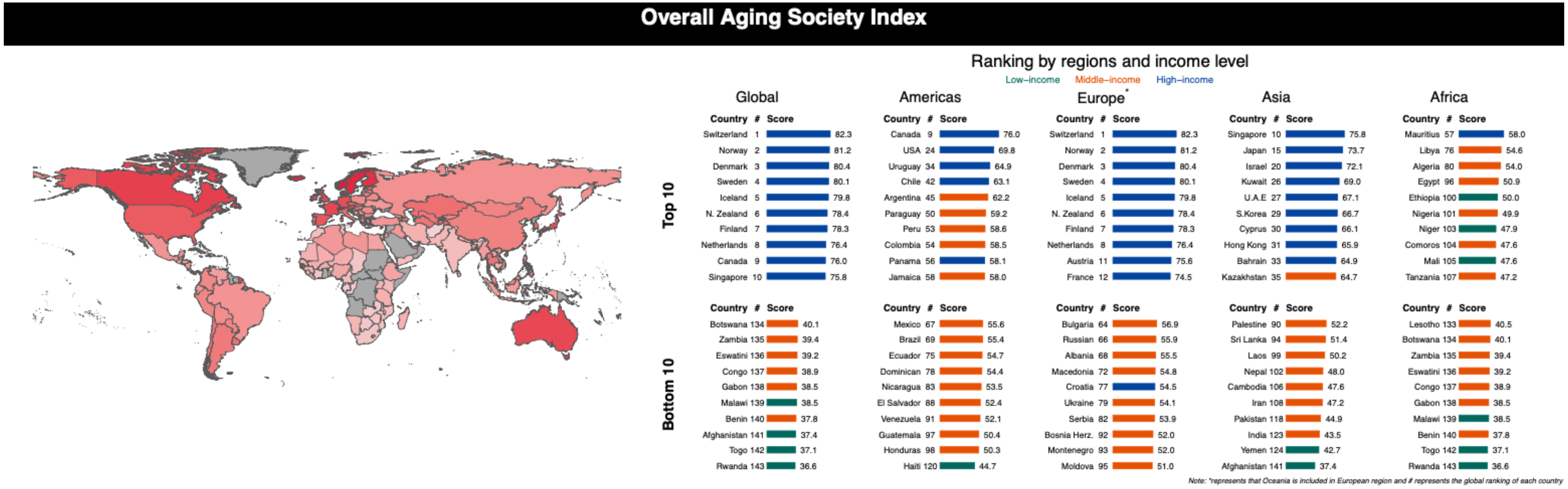

Supplementary table S4. Number of Countries, by Income Categories, for the Global aging society index and other indices.

	Global Aging Society Index	Active Aging Index (EU)	Global Agewatch Index	Human Development Index
Low	20	0	6	27
Middle	77	1	49	103
High	46	27	41	59
Total	143	28	96	189

Supplementary table S5. A comparison of the country specific assessments of global aging society index and the Human Development Index, in terms of number of measures in each domain

Domain	Global Aging Society Index	Human Development Index
Productivity and Engagement	5	0
Well Being	4	1
Equity	7	2
Cohesion	4	0
Security	5	1
Total	25	4

Supplementary Table S6. Domain and Index Rankings of Countries

Country	Overall Ranking	Productivity Engagement Ranking	Well Being Ranking	Equity Ranking	Cohesion Ranking	Security Ranking
Switzerland	1	1	3	9	25	2
Norway	2	3	17	1	23	1
Denmark	3	6	9	4	10	3
Sweden	4	4	5	5	21	11
Iceland	5	15	2	3	3	19
New Zealand	6	2	18	7	9	28
Finland	7	12	8	2	31	9
Netherlands (the)	8	5	10	10	41	22
Canada	9	9	14	13	17	30
Singapore	10	17	1	36	42	6
Austria	11	14	15	18	26	8
France	12	16	7	23	32	18

Australia	13	7	20	14	7	51
United Kingdom of Great Britain and Northern Ireland (the)	14	11	23	19	20	26
Japan	15	29	4	27	30	10
Ireland	16	21	11	20	5	36
Germany	17	13	22	16	55	14
Malta	18	33	13	38	14	5
Slovenia	19	37	32	6	33	4
Israel	20	25	6	33	37	24
Luxembourg	21	26	12	35	36	15
Belgium	22	20	28	17	27	31
Spain	23	27	16	54	4	32
United States of America (the)	24	8	96	15	28	16
Estonia	25	43	35	8	35	37
Kuwait	26	28	26	39	13	46
United Arab Emirates (the)	27	19	74	28	2	58
Czechia	28	52	49	11	69	27
Korea (the Republic of)	29	57	19	42	104	17
Cyprus	30	31	31	37	101	29
Hong Kong	31	51	33	46	46	38
Italy	32	54	21	43	63	45
Bahrain	33	49	53	45	1	63
Uruguay	34	58	30	65	34	39
Kazakhstan	35	81	75	12	11	47
Tajikistan	36	119	62	26	15	7
Hungary	37	48	68	41	52	25
Thailand	38	41	41	108	22	23
Slovakia	39	71	51	21	39	55
Portugal	40	50	36	61	47	52
Turkmenistan	41	104	48	32	12	41
Chile	42	23	40	74	49	65
Uzbekistan	43	124	46	55	8	12
Latvia	44	70	47	31	56	50

Argentina	45	59	43	73	44	48
China	46	108	50	83	51	13
Mongolia	47	102	65	71	16	43
Azerbaijan	48	132	70	34	29	35
Lithuania	49	93	52	29	91	61
Paraguay	50	10	63	120	43	90
Kyrgyzstan	51	99	44	30	24	96
Greece	52	109	29	59	78	70
Peru	53	18	24	106	124	88
Colombia	54	63	27	129	84	53
Philippines (the)	55	32	86	87	73	59
Panama	56	35	38	99	65	89
Mauritius	57	76	78	100	58	34
Jamaica	58	55	37	107	89	76
Poland	59	116	42	40	68	72
Belarus	60	129	59	22	106	44
Costa Rica	61	36	25	95	50	113
Bolivia (Plurinational State of)	62	30	76	91	130	40
Romania	63	135	45	69	99	21
Bulgaria	64	88	81	85	71	42
Malaysia	65	74	85	92	38	67
Russian Federation (the)	66	118	84	56	80	49
Mexico	67	45	77	127	81	60
Albania	68	125	34	79	110	54
Brazil	69	62	54	128	62	73
Armenia	70	128	56	57	95	56
Turkey	71	120	61	112	88	20
North Macedonia	72	112	87	48	97	62
Georgia	73	136	92	62	77	33
Viet Nam	74	98	67	76	75	78
Ecuador	75	39	57	113	125	74
Libya	76	106	80	101	6	83

Croatia	77	131	69	63	54	69
Dominican Republic (the)	78	72	39	114	48	107
Ukraine	79	126	91	50	87	57
Algeria	80	133	71	25	61	95
Iraq	81	140	102	24	18	81
Serbia	82	123	60	67	79	75
Nicaragua	83	46	58	126	70	103
Lebanon	84	111	82	53	19	109
Indonesia	85	22	117	86	100	101
Bangladesh	86	90	99	51	90	99
Myanmar	87	100	114	47	57	91
El Salvador	88	56	55	117	126	92
Jordan	89	143	64	81	53	64
Palestine, State of	90	138	105	52	59	66
Venezuela (Bolivarian Republic of)	91	73	79	135	45	87
Bosnia and Herzegovina	92	139	73	49	85	82
Montenegro	93	122	72	82	93	77
Sri Lanka	94	66	109	110	74	84
Moldova (the Republic of)	95	107	66	44	117	108
Egypt	96	137	104	58	86	68
Guatemala	97	40	83	123	115	106
Honduras	98	61	97	134	92	79
Lao People's Democratic Republic (the)	99	94	108	72	96	97
Ethiopia	100	67	112	75	76	112
Nigeria	101	53	107	64	105	123
Nepal	102	115	118	97	67	94
Niger (the)	103	85	101	66	98	129
Comoros (the)	104	82	93	105	108	117
Mali	105	101	95	78	72	136
Cambodia	106	75	122	111	103	104
Tanzania, United Republic of	107	47	123	102	111	114
Iran (Islamic Republic of)	108	127	89	109	116	85

Burkina Faso	109	80	110	89	109	115
Liberia	110	34	120	94	66	143
Mauritania	111	105	90	68	94	138
Gambia (the)	112	86	103	88	60	140
Madagascar	113	42	115	98	114	134
Guinea	114	79	111	77	107	135
Senegal	115	97	98	96	102	126
Uganda	116	69	125	118	64	124
Namibia	117	64	136	137	83	98
Pakistan	118	95	137	70	82	122
Tunisia	119	141	88	90	123	105
Haiti	120	38	113	124	138	119
Kenya	121	24	128	131	132	120
Cameroon	122	60	124	119	134	116
India	123	84	141	80	127	93
Yemen	124	142	126	84	40	131
Morocco	125	134	94	103	133	110
Mozambique	126	44	133	141	120	118
Sierra Leone	127	92	130	93	119	142
Zimbabwe	128	91	138	132	118	102
South Africa	129	130	129	142	122	80
Chad	130	83	134	104	128	139
Ivory Coast	131	96	100	125	135	137
Burundi	132	68	131	115	142	111
Lesotho	133	78	142	116	112	100
Botswana	134	89	140	136	139	71
Zambia	135	65	135	139	129	128
Eswatini	136	117	139	143	113	86
Congo (the)	137	87	116	140	137	125
Gabon	138	121	119	121	140	121
Malawi	139	77	127	130	141	130
Benin	140	114	106	122	143	132

Afghanistan	141	103	143	60	121	133
Togo	142	113	121	133	131	141
Rwanda	143	110	132	138	136	127

Supplementary Table S7: Percentage of missingness by measures and income level

**Countries with missing data (%)**	**All countries (n = 143)**	**Low income (n = 20)**	**Middle Income (n = 77)**	**High Income (n = 46)**
Labor force participation rate	0%	0%	0%	0%
Retraining	36%	65%	43%	13%
Volunteer	1%	0%	0%	2%
Work Quality	22%	45%	27%	2%
Active And Productive	4%	5%	6%	0%
HALE	2%	0%	1%	4%
HALE/LE	2%	0%	1%	4%
Life Satisfaction	1%	5%	0%	0%
UHC	1%	0%	1%	2%
Gini Coefficient	6%	5%	3%	11%
Poverty	0%	0%	0%	0%
Food Security	1%	0%	0%	2%
High School	34%	80%	42%	2%
Trust Neighbours	6%	5%	6%	7%
Social Support	0%	0%	0%	0%
Co-residence	11%	5%	12%	13%
Technology	0%	0%	0%	0%
Income	35%	60%	43%	11%
Pension	2%	5%	3%	0%
Safety	1%	0%	0%	2%
Quality of Healthcare	1%	0%	1%	0%
No Mental Health	6%	15%	4%	7%

Supplementary Figure S8: Sensitivity scatterplots around missing data

Mean vs. 25th percentile imputation	Mean vs. 75th percentile imputation	ALL Measures vs. Only measures with less than 10% missing data (using mean imputation)

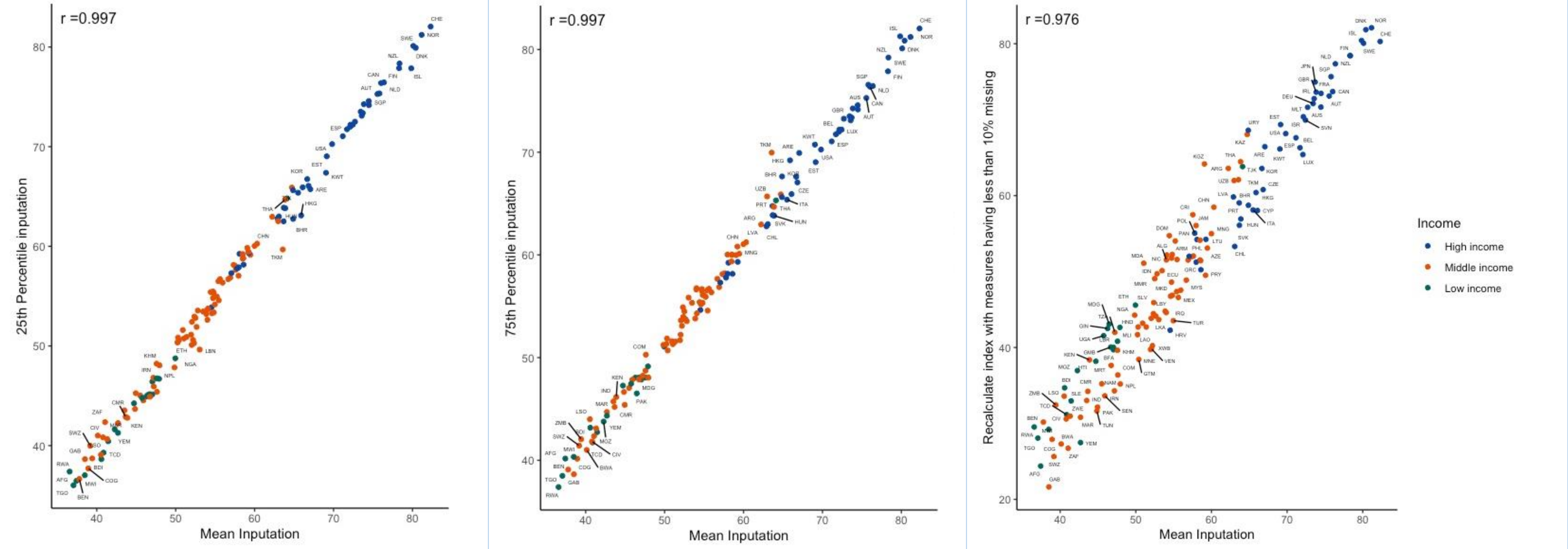

Supplement: Supplementary file 1 — Supplementary Figs. 1–7 and Tables 1–7. [file 43587_2024_772_MOESM1_ESM.pdf]
